# Neutrophil extracellular traps and their histones promote Th17 cell differentiation directly via TLR2

**DOI:** 10.1038/s41467-022-28172-4

**Published:** 2022-01-26

**Authors:** Alicia S. Wilson, Katrina L. Randall, Jessica A. Pettitt, Julia I. Ellyard, Antje Blumenthal, Anselm Enders, Benjamin J. Quah, Tobias Bopp, Christopher R. Parish, Anne Brüstle

**Affiliations:** 1 The John Curtin School of Medical Research, The Australian National University, Canberra, ACT Australia; 2 Institute for Immunology, University Medical Center, Johannes Gutenberg University Mainz, Mainz, Germany; 3 ANU Medical School, The Australian National University, Canberra, ACT Australia; 4 The University of Queensland Diamantina Institute, The University of Queensland, Brisbane, QLD Australia

**Keywords:** Neutrophils, T-helper 17 cells

## Abstract

Neutrophils perform critical functions in the innate response to infection, including through the production of neutrophil extracellular traps (NETs) - web-like DNA structures which are extruded from neutrophils upon activation. Elevated levels of NETs have been linked to autoimmunity but this association is poorly understood. By contrast, IL-17 producing Th17 cells are a key player in various autoimmune diseases but are also crucial for immunity against fungal and bacterial infections. Here we show that NETs, through their protein component histones, directly activate T cells and specifically enhance Th17 cell differentiation. This modulatory role of neutrophils, NETs and their histones is mediated downstream of TLR2 in T cells, resulting in phosphorylation of STAT3. The innate stimulation of a specific adaptive immune cell subset provides an additional mechanism demonstrating a direct link between neutrophils, NETs and T cell autoimmunity.

## Abstract (web summary)

Neutrophils are critical in the immune response to infective agents and have multiple effector strategies including the production of extracellular traps termed NETs. Here the authors show a link between NET production and Th17 differentiation which mechanistically occurs downstream of TLR2 signalling.

## Introduction

Neutrophils are well known for their effective and instant killing of bacteria in tissues but the discovery of neutrophil extracellular traps (NETs) has widened our knowledge of their importance, particularly with regards to their potential systemic influences. Whilst originally discovered for their role in capturing and killing bacteria during infections^1^, our understanding of the involvement of NETs in infection and immunity is rapidly expanding. To date, NETs have been associated with bacterial, fungal, viral, and parasitic infections as well as many autoimmune conditions, including systemic lupus erythematosus^2^ and rheumatoid arthritis^3^. While their involvement in the clearance of infection is relatively well understood, whether they are an active effector or a product of autoimmune disease is still unclear.

NETs contain large numbers of DNA-bound antimicrobial proteins derived from the neutrophil cytoplasm and nucleus that are released from neutrophils upon stimulation. Notably, histones, (specifically H2A, H2B, H3, and H4), highly conserved, small, cationic proteins which support the nuclear condensation of DNA, form up to 70% of NET-bound proteins^4^. In vivo, histones can be found either surrounded by DNA or in DNA-free octameric protein form^5^. This release of free histones from NETs can be mediated by endogenous and pathogen-derived DNases. DNA-free histones are much more pathological than their DNA-bound counterparts and have been implicated as mediators of tissue damage in sepsis, acute organ injury, and trauma^6–8^. Interestingly, it has been recently suggested that histones are proinflammatory at sub-cytotoxic levels^9^. Along these lines, elevated extracellular histone levels have also been detected in multiple autoimmune and inflammatory diseases, including atherosclerosis^10^ and rheumatoid arthritis (RA)^11^. Whilst the presence of increased NETs and histones in autoimmune diseases is well known, the functional relevance of NETs and their components in these conditions is unknown.

The response of neutrophils to bacterial and fungal infections can be accelerated by an adaptive immune response to these pathogens. The IL-1-producing CD4^+^ T helper cell subset Th17 cells is well described as a potent inducer of neutrophils^12^ as such, Th17 cells are critical for immunity against *Candida albicans* and *Staphylococcus aureus* infections^13^. The differentiation of naive T cells into Th17 cells requires an IL-6 and TGF-β rich environment^14^. Alterations to the cytokine microenvironment, such as the presence of IL-23 and myeloid-derived IL-1β can promote Th17 cell generation and alter their effector functions^15,16^. The induction of transcription factor RORγt is a key part of the transcriptional programming required for Th17 cell differentiation and effector functions such as the production of their namesake cytokine IL-17 and GM-CSF, which actively recruit neutrophils to sites of inflammation, promoting inflammatory processes^17,18^. Th17 cells have also been implicated in the pathogenesis of autoimmune diseases, including multiple sclerosis^19,20^ and RA^21,22^. The ability to mimic the differentiation microenvironment in vitro to induce the stable differentiation of Th17 cells is a key tool for investigating these complex cell–cell interactions.

While often considered as terminal effectors of Th17 cell function, the interaction between Th17 cells and neutrophils is bidirectional. Neutrophils secrete cytokines and chemokines which can directly influence T cell activation, differentiation, and recruitment and have even been shown to be able to present antigens^23,24^. While there is a clear association between autoimmunity and neutrophils, the contribution of NETs to the development of pathological CD4^+^ T cell responses has so far only been described through enhancement of the inflammatory microenvironment by stimulating innate immune cells^25,26^. Here, we elucidate a mechanism by which NETs can directly promote the generation of the proinflammatory Th17 cell subset demonstrating a feedback loop between innate and adaptive immunity which amplifies autoimmunity.

## Results

### NETs directly promote Th17 cell differentiation through histones, an effect ameliorated by a first-in-class histone inhibitor

**Fig. 1:**
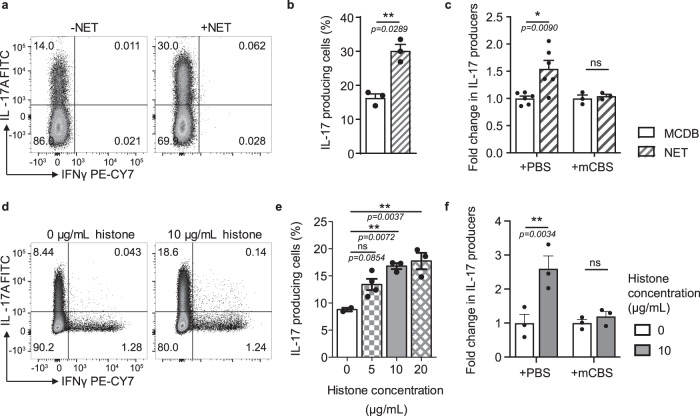
Neutrophil extracellular traps (NETs) promote Th17 cell differentiation through histones. **a**, **b** IL-17 and IFNγ expression by in vitro differentiated Th17 cells measured by flow cytometry after culture in the presence or absence of in vitro generated NETs *n* = 3. **c** Normalized IL-17 production by Th17 cells differentiated in the presence of NETs in MCDB media or MCDB media alone with the addition of histone inhibitor mCBS. **d**, **e** IL-17 and IFNγ expression by in vitro differentiated Th17 cells cultured with a low concentration of histones (0–20 µg/mL) *n* = 3. **f** Normalized IL-17 production of Th17 cells differentiated with histones with the addition of histone inhibitor mCBS *n* = 3. Data bars show mean ± SEM. “ns” (not significant), *p* > 0.05; “*”*p* < 0.05; “**”*p* < 0.01. **b** Paired two-tailed *t*-test, **c**, **f** two-way ANOVA with Sidak’s multiple comparisons test, **e** one-way ANOVA with Tukey’s multiple comparisons test. Data points represent individual mice. All data are representative of a minimum of two independent experiments. Source data are provided as a Source Data file.

To determine whether NETs directly affect Th17 cell differentiation, naive T cells were co-cultured with in vitro generated NETs in the presence of the Th17 cell-promoting cytokines, IL-6, and TGF-β. The presence of NETs during Th17 cell differentiation induced a strongly enhanced IL-17 response when compared to the media-only control (Fig. 1a, b). While the protein composition of NETs varies across species, histones are a highly conserved and the most abundant protein component of NETs^4^. To determine whether there was a role for NET-bound histones in NET-induced Th17 cell induction, β-methyl-cellobioside sulfate (mCBS), a recently identified, first-in-class inhibitor of both NET-bound and free histone cytotoxicity^27^ was added to NET-T cell co-cultures. The specific inhibition of histones by mCBS ameliorated the enhancing effect of NETs on Th17 cells (Fig. 1c).

To investigate whether indeed histones are responsible for the promotion of Th17 cell differentiation by NETs, we examined the effects of free histone octamers on Th17 cell differentiation. Free histones similar to NETs promoted the enhanced production of IL-17 in a dose-dependent manner (Fig. 1d, e). While histones have been described to induce cell death^6^, notably the Th17 cell enhancing concentrations did not induce any histone-related cell death (Supplementary Fig. 1). To further confirm that the enhancement of Th17 cell differentiation is histone-dependent, the histone inhibitor mCBS was added to histone-T cell co-cultures. The addition of mCBS completely abolished the promotion of cytokine production (Fig. 1f). Our findings, therefore, implicate histones as the driving elements behind the NET-induced increase in Th17 cell differentiation.

### Histones specifically promote Th17 cell generation in vivo

**Fig. 2:**
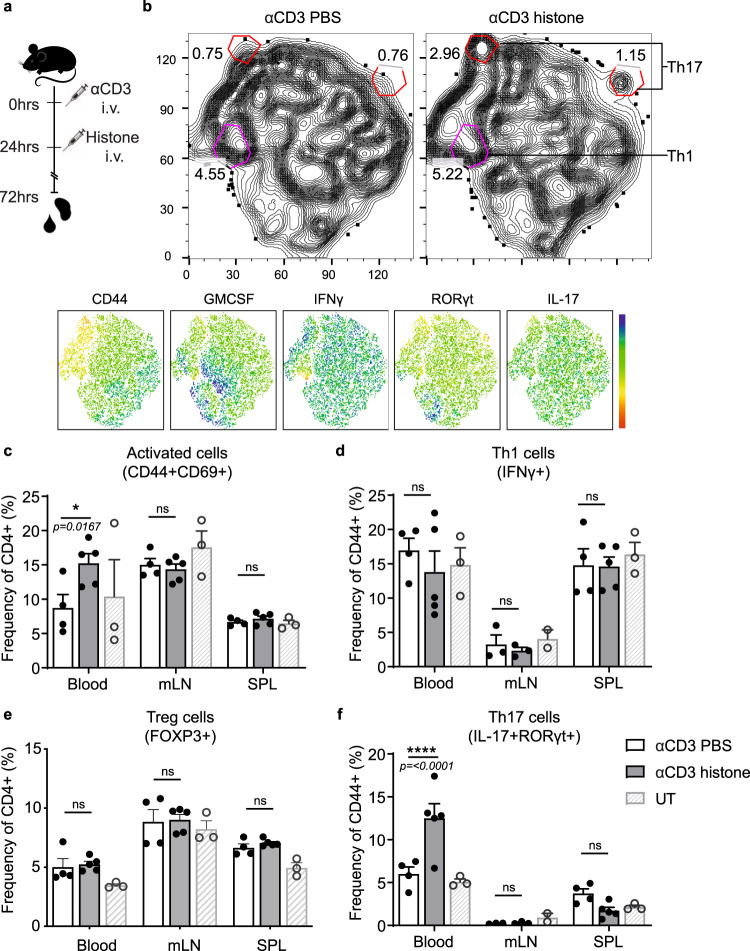
Histone induction of Th17 cell response is conserved in vivo. **a** Histones were injected intravenously 24 h after injection of low dose αCD3 and 48 h later blood and lymphoid organs were analyzed. **b** TsNE analysis of flow cytometric data of CD4^+^ T cells with clustering based on the production of cytokines and expression of CD44. Th17 cell populations (top red circles) and Th1 cell populations (bottom pink circle) are highlighted. Quantification of **c** activated CD4^+^ T cells, **d** Th1, **e** Treg, and **f** Th17 cells based on conventional gating of flow cytometry data was also performed. Data bars show mean ± SEM. “ns” (not significant), *p* > 0.05; “*”*p* < 0.05; “****”*p* < 0.0001. **c**–**e** Two-way ANOVA with Dunnett’s multiple comparisons test. Data points represent individual mice. All data are representative of a minimum of two independent experiments. Source data are provided as a Source Data file.

To determine if this histone-dependent effect observed in vitro had relevance in vivo, we investigated their effect in a mouse model. Mice were given a low dose of anti-CD3 (0.1 μg/g anti-CD3) intravenously, and 24 h later given either 1 μg/g histone or PBS i.v. Forty-eight hours after the second injection, blood, spleen, mesenteric lymph nodes, and Peyer’s patches were analyzed for cell viability and T cell activation (Fig. 2a). TsNE analysis of cytokine production of CD4^+^ T cells from the blood of dosed mice showed a selective overrepresentation of populations of Th17-like (IL-17^+^RORγt^+^) cells (Fig. 2b). Histone injection after anti-CD3 administration also led to a significant increase in activated (CD44^+^CD69^+^) CD4^+^ T cells in the blood of mice (Fig. 2c and Supplementary Fig. 2a). While no changes were seen in the production of the Th1 cell-associated cytokine, IFNγ, or in regulatory T (Treg) cell frequencies in any organ after histone injection (Fig. 2d, e and Supplementary Fig. 2a), treatment with histones induced a more than two-fold increase in Th17 (RORγt^+^IL-17^+^) cells in the blood of mice (Fig. 2f) confirming observations from TsNE analysis. Further, Th17 cells but not Th1 nor Treg cells from mice receiving histones expressed higher levels of CD44 than PBS controls reflecting enhanced activation (Supplementary Fig. 2d). This effect was independent of cell death (Supplementary Fig. 2b, c) and generalized increases in Th17 promoting cytokines (Supplementary Fig. 2e), but dependent on TCR engagement (Supplementary Fig. 2f), indicating an in vivo role for histones in promoting a rapid Th17 cell-specific response.

### Histones specifically induce Th17 cell differentiation through RORγt

**Fig. 3:**
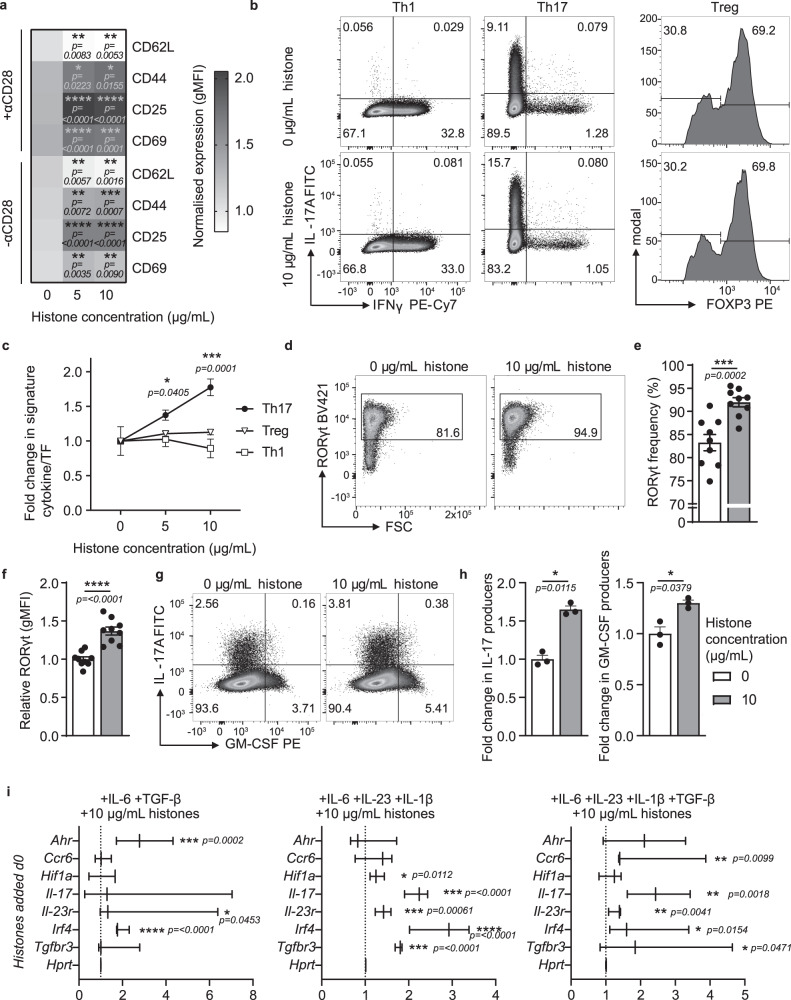
Histones induce a Th17 cell-specific response. **a** Mean expression levels of T cell activation markers after 16 h co-culture with 0–10 µg/mL histone in the presence or absence (±) of αCD28 measured by flow cytometry *n* = 5. gMFI of each marker was normalized to the mean of no αCD28, 0 µg/mL condition and heat map created from the mean of the normalized values. **b** Differentiation of mouse naive T cells into Th1, Th17, and Treg cells in the presence of histones was measured by flow cytometric quantification of the expression of key factors IFNγ, IL-17 and FOXP3 from total live cells. **c** Graphical representation of **b** at various concentrations of histones with expression levels normalized to expression levels in the absence of histones; Th17 10 µg/mL histones *n* = 3, all others *n* = 4. **d**, **e** Frequency of cells expressing RORγt and **f** relative total RORγt expression at 32 h (calculated from the geometric mean) measured by flow cytometry *n* = 9. **g**, **h** Differentiation of mouse naive T cells into “pathogenic” Th17 cells with the addition of IL-23 in the presence of histones measured by flow cytometric quantification of IL-17 and GM-CSF after 5 days of culture *n* = 3. **i** Relative gene expression (fold-change) of histone-treated non-pathogenic (left) and pathogenic Th17 cells ± TGFβ after 3 days of differentiation compared to H_2_O-treated controls. *Hprt* expression was used as a housekeeping control *n* = 3. **c**, **e**, **f**, **h** Data bars show mean ± SEM. or **i** range and distribution, *p* > 0.05; “*”*p* < 0.05; “**”*p* < 0.01; “***”*p* < 0.001; “****”*p* < 0.0001. Where significance is not indicated, differences were ns. **a**, **c** Two-way ANOVA with Tukey’s multiple comparisons test, **e**, **f**, **h** paired student’s two-tailed *t*-test, **i** one-sample *t*-test (performed on ΔΔC*t* values). Data points represent individual mice. All data are representative of a minimum of two independent experiments. Source data are provided as a Source Data file.

To understand the in vivo adaptive immune response to histones we investigated the direct effect of histones on T cells in culture. Given the increased activation of T cells observed in response to histones in vivo, naive CD4^+^ T cells were incubated with histones and the expression of surface markers of activation was measured. Activation of naive CD4^+^ T cells was observed in response to treatment with a low concentration of histones even in the absence of co-stimulation (Fig. 3a and Supplementary Fig. 3a, b).

Our in vivo data indicate that histones specifically promote the development of Th17 cells, but not other T cell subsets tested. To determine if the effect was indeed specific to Th17 cells, we investigated the differentiation of naive CD4^+^ T cells into different T cell subsets strongly associated with autoimmunity in the presence of histones in vitro. The differentiation of naive CD4^+^ T cells into Th1 (as determined by the frequency of cells expressing the Th1 signature cytokine IFNγ) and Treg cells (as measured by expression of the transcription factor FOXP3) (Fig. 3b, c), was not affected in the presence of histones. This further indicates that the presence of histones in T cell cultures specifically drives Th17 cell differentiation. Concordantly, the analysis of RORγt expression of cells after histone treatment showed that histones induced both an increase in the frequency of RORγt expressing cells and an increase in the per-cell expression of RORγt early in the differentiation process (Fig. 3d–f), before all cells reached saturation of RORγt expression at 48 h (Supplementary Fig. 4a). This specific increase in Th17 cell populations was also observed under pathogenic Th17 cell differentiation conditions (Fig. 3g) where both IL-17 and GM-CSF production were increased in the presence of histones (Fig. 3h) supporting the idea of an overall enhancement of Th17 cell differentiation rather than an isolated induction of IL-17. Furthermore, Th17 cell cultures showed an increase in proliferation in response to histones with cells undergoing slightly more divisions in the presence of histones (Supplementary Fig. 4b, c).

To further examine the effects of histones on Th17 cell differentiation, we analyzed the expression of a set of Th17 cell-associated genes in cells cultured under non-pathogenic and pathogenic Th17 cell inducing conditions. Cells cultured with histones under Th17 cell differentiating conditions showed increased expression of Th17 cell-associated genes. Notably, upregulation of *Irf4* (Fig. 3i) which is both necessary for Th17 cell induction^28^ and implicated in the regulation of IL-17 promoter activity^29^ and *Il-23r*, a gene associated with Th17 cell pathogenicity^15^, was seen in response to histone co-culture (Fig. 3i). The addition of histones after 3 days of culture likewise led to increased expression of Th17 cell-associated genes in both pathogenic and non-pathogenic Th17 cell subsets (Supplementary Fig. 4d).

### Histone promotion of Th17 cells is conserved in human cells

**Fig. 4:**
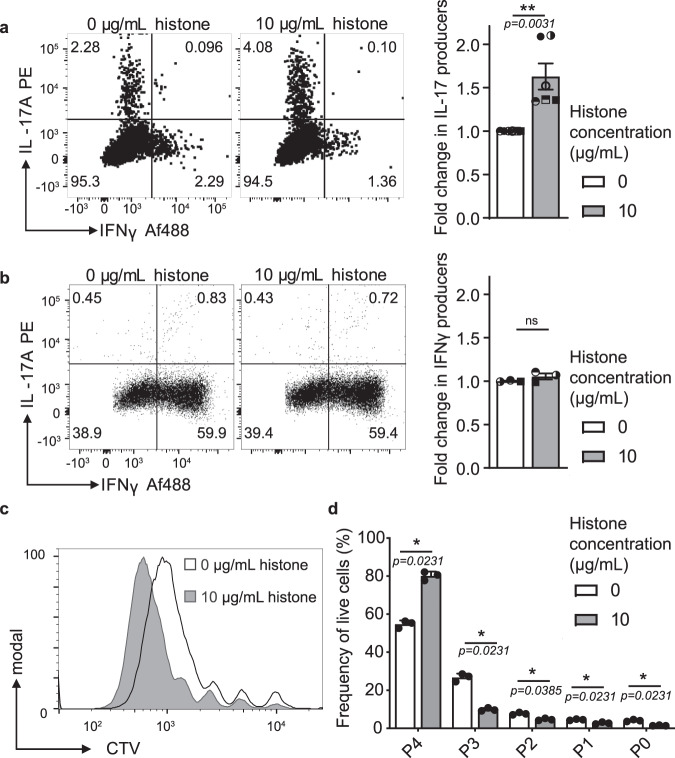
Histone-induced promotion of Th17 cell differentiation is conserved in humans. Differentiation of human **a** Th17 cells and **b** Th1 cells under differentiating conditions in the presence or absence of histones for 6 days was measured by IL-17 and IFNγ production by flow cytometry. Key cytokine production of each subset was normalized to the 0 µg/mL histone condition for each donor *n*(a) = 6, *n*(b) = 3. **c** Proliferation of Th17 cell cultures in the presence or absence of histones was measured by dilution of CTV using flow cytometry and **d** peaks indicating cell divisions quantified *n* = 3. Data bars show mean ± SEM. “ns” (not significant), *p* > 0.05; “*”*p* < 0.05; “**”*p* < 0.01. **a**, **b** Paired two-tailed *t*-test **d** two-tailed *t*-test with Holm–Sidak correction. Data points represent individual donors with data pooled from multiple independent experiments. Source data are provided as a Source Data file.

We next wondered if these effects are specific to the murine systems used or if the high homology of histones between mouse and human is an indication for a mechanism conserved between species. To demonstrate that we first confirmed the limited cytotoxicity of low concentrations of histones on human peripheral blood mononuclear cells (PBMCs) (Supplementary Fig. 5a). Human naive T cells were then isolated from PBMCs from healthy donors and differentiated under Th17 cell differentiation conditions in the presence of low concentrations of histones. Histones enhance the IL-17 production of human T cells under Th17 inducing conditions (Fig. 4a) in a manner comparable to that seen in mouse cell cultures. Similarly to murine differentiation, this was observed to be a Th17 cell-specific effect as the addition of histones to human Th1 cell differentiation cultures had no impact on the production of the Th1 signature cytokine IFNγ (Fig. 4b). Alongside the increase in the frequency of cells producing IL-17 in Th17 cell differentiation cultures (Fig. 4a), an increase in both Th17 and Th1 cell proliferation was also observed with the addition of histones (Fig. 4c, d and Supplementary Fig. 5b, c). Interestingly, increases seen in IL-17 expression in Th17 cell cultures were uncoupled from increased proliferation hinting at independent pathways (Supplementary Fig. 5d). These data cumulatively indicate not only an induction of cytokines but an additional increase in Th17 cell numbers in response to histones.

### Histones drive Th17 cell responses through a TLR2/MyD88-dependent pathway

Both the highly conserved nature of histones and the cross-species effects observed argue for an evolutionarily conserved histone receptor on T cells. Histones have previously been shown to engage toll-like receptors (TLRs) 2 and 4 on innate immune cells and epithelial cells^30,31^. These TLRs which are part of a family of highly conserved pattern recognition receptors^32^ have also been independently shown to impact T cell differentiation^33^. This correlational evidence suggests that TLRs could be potential mediators of histone-driven Th17 cell differentiation.

**Fig. 5:**
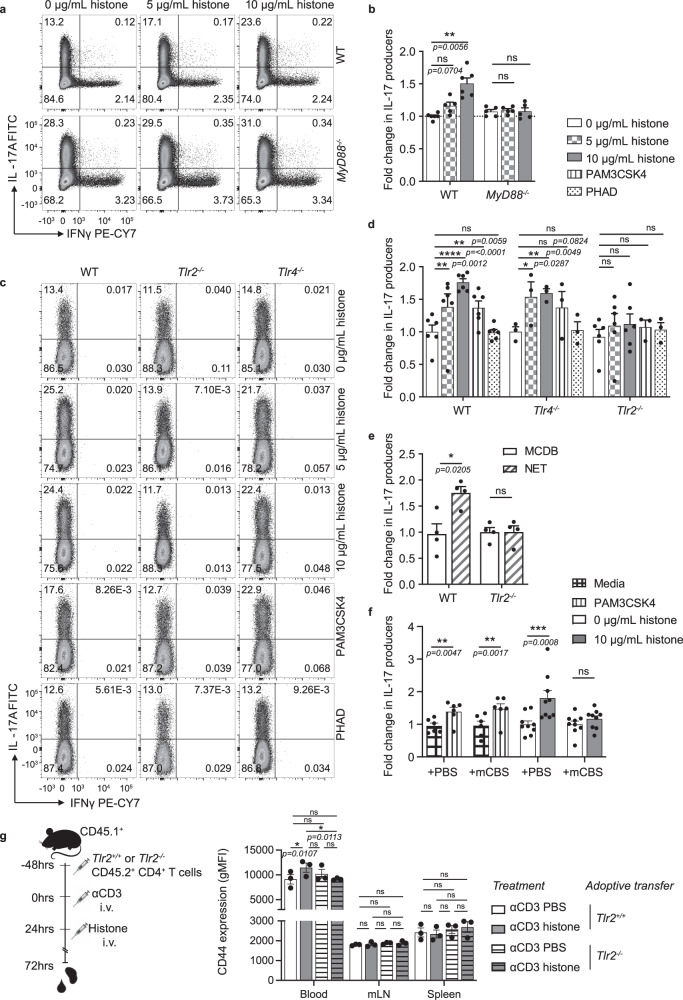
Histone-induced Th17 cell differentiation is dependent on MyD88 and TLR2. **a** Flow cytometric analysis and **b** quantification of relative IL-17 production in response to histone treatment of *MyD88*^*−/−*^ and WT T cells from littermate mice *n*(WT) = 6, *n*(*MyD88*^*−/−*^) = 5. **c** Flow cytometry plots and **d** quantified fold-change in IL-17 expressing cells after addition of histones, TLR2 and TLR4 agonists (PAM3CSK4 and PHAD, respectively) to Th17 cell differentiation cultures of naive T cells from *Tlr4*^*−/−*^ and Tlr*2*^*−/−*^ mice *n*(WT) = 6, *n*(*Tlr4*^*−/−*^) = 3, *n*(*Tlr2*^*−/−*^) = 5. **e** Fold-change in IL-17 production of differentiated Th17 cell cells in response to co-incubation with NETs in TLR2 KO mice *n*(WT) = 8, *n*(*Tlr2*^*−/−*^) = 4. **f** Relative IL-17 expression after Th17 cell differentiation in the presence of PAM3CSK4 or histones in the presence or absence of histone inhibitor mCBS. **g** CD4^+^ T cells isolated from *Tlr2*^*+/+*^ and *Tlr2*^*−/−*^ mice were injected in C57Bl/6 CD45.1 congenic recipient mice 48 h before low dose αCD3 and 72 h before intravenous histone injections. CD45.2 congenic transferred cells were analyzed 48 h after histone injection for CD44 expression *n* = 3. Data bars show mean ± SEM. “ns” (not significant), *p* > 0.05; “*”*p* < 0.05; “**”*p* < 0.01; “***”*p* < 0.001; “****”*p* < 0.0001. Where significance is not indicated, differences were ns. **b**, **e**, **f**, **g** Two-way ANOVA with Sidak’s correction, **d** mixed-effects model (REML). Normalized data are shown to allow pooling of experimental data, with all data representative of a minimum of two independent experiments. Data points represent individual mice. Source data are provided as a Source Data file.

T cell differentiation of Th17 cells with histones was performed on naive CD4^+^ T cells isolated from mice lacking MyD88; a key adaptor molecule in signaling through most TLRs including both TLR2 and TLR4^34^. The increase in Th17 cell differentiation by histones was abolished in the absence of MyD88, indicating a TLR, MyD88-dependent mechanism (Fig. 5a, b).

To determine whether stimulation of TLR2 or TLR4 was sufficient to drive increased Th17 cell differentiation, we differentiated Th17 cells in the presence of histones, the TLR2 agonist PAM3CSK4 or the TLR4 agonist PHAD. While PAM3CSK4 promoted the production of IL-17 (Fig. 5c, d), the TLR4 agonist PHAD did not promote Th17 differentiation (Fig. 5c, d). PHAD activity was confirmed by HEK-Blue™ mTLR4 cells (Supplementary Fig. 6a), while another TLR2 agonist, PAM2CSK4 was also able to promote IL-17 production by Th17 cells (Supplementary Fig. 6b). The differentiation of naive T cells from TLR2- and TLR4-deficient mice showed that histones were able to promote differentiation of Th17 cells in the absence of TLR4, whilst the absence of TLR2 completely abolished this effect (Fig. 5c, d). Similarly, the absence of TLR2 rendered T cells unable to respond to in vitro generated NETs (Fig. 5e). To rule out the possibility that our observed effects were due to TLR2 ligand contamination of the standard, commercially available, histone preparation used, we stimulated Th17 cells with TLR2 ligand PAM3CSK4 in the presence or absence of the histone inhibitor mCBS. While mCBS was able to abolish the increased IL-17 production resulting from the addition of histones (Fig. 5f), it did not alter the effect of PAM3CSK4. Additionally, the exposure of differentiating Th17 cells to DNA rich in CpG did not promote IL-17 expression (Supplementary Fig. 6c), further indicating the histone proteins as the driving force behind the increase in IL-17 production.

To confirm the relevance of these effects using our model of in vivo histone delivery, *Tlr2*^+/+^ or *Tlr2*^−^^/−^ CD4^+^ T cells were adoptively transferred into congenically labeled recipient mice before delivery of low dose anti-CD3 and subsequent administration of histones (Fig. 5g). While cell subsets were not able to be discerned due to low cell number, cell activation, measured 48 h after histone delivery showed that, in response to histones, only transferred *Tlr2*^+/+^ cells, not their *Tlr2*^−^^/−^ counterparts displayed increased activation (as measured by CD44 expression) in the blood (Fig. 5g). This effect was independent of proliferation, with equal frequencies of transferred cells recovered between all groups (Supplementary Fig. 6e). Taken together, our findings demonstrate that histones specifically activate TLR2 on CD4^+^ T cells to promote activation and differentiation into Th17 cells.

### Histone-TLR2 interactions induce STAT3 phosphorylation

**Fig. 6:**
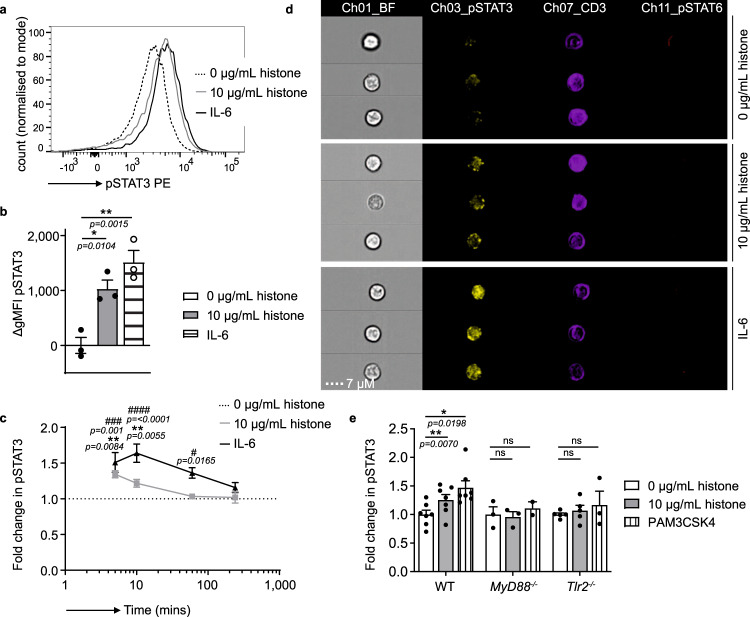
Histones induce rapid phosphorylation of STAT3 in a MyD88-, TLR2-dependent manner. **a** Histogram showing the geometric mean of fluorescence intensity (gMFI) and **b** quantification of the change in (Δ) the gMFI of tyrosine 705 phosphorylation of STAT3 (pSTAT3) after 5 min incubation with histones (10 µg/mL, gray line) or IL-6 (black line) compared to controls (dashed black line) *n* = 3. **c** Relative gMFI of pSTAT3 compared to matched 0 µg/mL control over time, *n* = 4–6 mice per time point. **d** Phosphorylation of STAT3 and STAT6 after 5 min of stimulation analyzed by imaging flow cytometry with images shown representative of the mean pSTAT3 fluorescence intensity after stimulation. **e** Relative gMFI of treated cells from *Myd88*^*−/−*^ and *Tlr2*^*−/−*^ mice after 5 min *n*(WT) = 8, *n*(*My88*^*−/−*^, *Tlr2*^*−/−*^) = 3. Data bars show mean ± SEM. “ns” (not significant), *p* > 0.05; “*/^#^”*p* < 0.05; “**”*p* < 0.01; “^###^”*p* < 0.001; “^####^”*p* < 0.0001. Where significance is not indicated, differences were ns. **b** One-way ANOVA with Dunnett’s multiple comparisons test, **c** mixed-effects model (REML) (where * denotes differences between 10 and 0 µg/mL controls, # denotes significance between IL-6 and 0 µg controls), **e** two-way ANOVA with Dunnet’s multiple comparisons test. All data are representative of a minimum of two independent experiments. Source data are provided as a Source Data file.

To understand how histones promote the induction of the Th17 cell-specific transcription factor RORγt, described previously (Fig. 3d–f), we examined phosphorylation of the key signaling transducer STAT3. The phosphorylation of STAT3 is a modification that is essential for Th17 cell formation and RORγt expression^35^. After a five-minute stimulation, naive T cells exposed to histones had increased phosphorylation of STAT3, similar to cells treated with the canonical STAT3 inducer IL-6 (Fig. 6a–c). This finding was confirmed using imaging flow cytometry (Fig. 6d and Supplementary Fig. 7). These effects were not due to a generalized phosphorylation of STAT proteins, as STAT6 phosphorylation was not induced by histones or IL-6 (Fig. 6d).

Most importantly, histones failed to induce STAT3 phosphorylation in the absence of MyD88 or TLR2 (Fig. 6e) further implicating histone engagement of TLR2 in driving Th17 cell responses. Additionally, the rapid phosphorylation of STAT3 after the addition of histones further emphasizes the direct link between histone binding and STAT3 phosphorylation. These data demonstrate a pathway where the direct binding of histones to TLR2 on T cells leads to the activation of STAT3 and RORγt and the subsequent promotion of Th17 cell cytokine production.

## Discussion

Our data show that histones, the major protein component of NETs, can directly interact with TLR2 expressed on T cells, directly inducing phosphorylation of STAT3, which in the presence of Th17 cell promoting cytokines, promotes Th17 cell differentiation and activity. This provides, for the first time, a mechanism to explain the long-standing finding that increased NETs are linked with T cell autoimmunity.

Engagement of TLR2 on T cells has previously been linked to Th17 cell differentiation^33^ however, the mechanism of how this occurred was unclear. Our data provide evidence that histones, signaling through TLR2 on T cells lead to the phosphorylation of STAT3 and subsequent induction of RORγt. While TLR2 has been associated with Th17 cells, the direct induction of Th17 cell-enhancing molecules such as STAT3 and RORγt has not been demonstrated previously.

The ablation of these signaling events in TLR2 and MyD88-deficient cells is clear evidence for this. One limitation of our study is the absence of direct visualization of the histone-TLR interaction opening the possibility of secondary rather than direct effects. However, this signaling occurs immediately after histone exposure with phosphorylation measurable after 5 min, strongly arguing against secondary effects. By using a highly controlled in vitro environment with flow cytometric sorted Th cells, we further excluded potential effects from contaminating TLR expressing APCs.

Further, while we show that NETs can directly influence Th17 cell differentiation, where this interaction may take place physiologically is unclear. As activation of naive T cells primarily occurs in secondary lymphoid organs, it is likely that the systemic increase in NET-derived free histones during infection and autoimmunity would allow the direct priming of Th17 cells at these sites. Separating the direct and indirect effects of NETs and their histones on the adaptive and innate immune systems is hard to achieve. In vitro cultures provide a clean and controlled environment that while having certain limitations, makes them a necessary tool to dissect these complex interactions. In fact, our experiments showing an effect of histones on T cell proliferation in vitro underline the essential role of in vitro assays to isolate a single factor from the complex interactions happening in vivo that completely mask this effect.

The absence of standard techniques for the quantification of both NETs and histones poses a challenge not only for their quantification in the lymph node but even in circulation leading to vast inconsistencies in the literature. Reported levels of “normal” circulating histones vary from undetectable levels^36^, to 0.06 ng/mL^37^ and even up to 3.6 μg/mL^6^. This becomes even more pronounced in a disease setting, for example, trauma where detected levels varied from 3 ng/mL^38^ to up to 230 μg/mL^6^. Further compounding this variability is the rapid uptake of small concentrations of circulating histones by the liver^39^. This clearing mechanism might explain why in the above described in vivo model only Th17 cells in circulation show a significant histone-induced increase in activation and IL-17 production.

Previous studies have described an indirect promotion of Th17 cell responses by NETs through macrophages^25^ and monocytes^26^. We here show that alongside these indirect effects, NETs and their histone components directly impact Th17 cell differentiation from naive precursors. Our findings demonstrate a convergence of the direct effects of NETs and indirect effects through stimulation of innate immune cells.

Given the ability of NETs and native nucleosomes to produce strong Th17 cell-inducing cytokine responses in the form of IL-6 and IL-1β production from myeloid cells^9,25,40^, the physiological relevance of the direct interaction of NETs and histones with Th17 cells needs to be addressed. We hypothesize that in conditions with high concentrations of circulating NETs and histones, such as in sepsis, indirect effects likely play a greater role than the direct effects demonstrated here. Low concentrations of histones, such as used here, only led to an increase in Th17 cell frequency and increased T cell activation in combination with T cell stimulation. Importantly, this occurred in the absence of increased concentrations of IL-1β or IL-6 as could be expected to be produced from myeloid cells in response to histones. This strongly suggests that in instances where there are low concentrations of circulating histones/NETs alongside weak TCR activation that direct interactions with T cells may play a larger role. The recently described ability of another neutrophil-derived factor, cathelicidin (LL-37/mCRAMP) to directly induce Th17 cell responses^41^ further supports the physiological relevance of these direct T cell interactions. The functional redundancy of these two pathways demonstrates the far-reaching impacts of NETs on the immune system and the specificity of the NET-Th17 cell response.

We have shown a previously undescribed, direct role of NETs in promoting an adaptive, proinflammatory immune response. Given recent evidence that IL-17 can directly induce neutrophil NET formation^42^, this could indicate that neutrophils through their NETs drive a feed-forward loop that, through the promotion of Th17 cell responses, drives further NET formation evolving neutrophils from terminal effectors to complex mediators. This data further explains the link between Th17 cells and neutrophil-mediated immunity that occurs such as in Candida albicans infection where Th17 cells are necessary for fungal clearance and the presence of NETs has also recently been described^4^.

Beyond the implications that these data hold for understanding beneficial neutrophil-Th17 interactions, these data will also have impacts upon pathogenic aspects of this feed-forward loop. The presence of extracellular histones and NETs has been described in sepsis^8^, COVID-19^43^, DVT^44^, atherosclerosis^10^, SLE^45^, RA^11^, and cancer^46^, with the levels of histones/nucleosomes correlated with severity in stroke and DVT [reviewed in ref. ^5^]. Indeed, the here used histone inhibitor, mCBS, is currently undergoing Phase 1b/2 clinical trials in sepsis and COVID-19 patients (personal communication and ref. ^27^), with infection-induced acute respiratory distress syndrome (ARDS), being one of the most serious complications in both sepsis and COVID-19 disease^47^. Th17 cells and their cytokines are known for their pathogenic/deleterious involvement in these conditions^48^ including COVID-19^47^ as well as in various cancers^49–51^. Our data show a putative feed-forward loop between NETs/histones and Th17 cells and gives an explanation of the abundance of NETs and Th17 cells in these conditions. Thus, interrupting this feed-forward loop using a direct histone inhibitor such as mCBS or through targeting of TLR2 provides an new potential therapeutic target for the treatment of these conditions.

## Methods

### Mice

Mice were housed in a pathogen-free facility and sex and age-matched controls were used for all experiments. All mice used were between 8 and 14 weeks of age, with littermates used unless otherwise stated. Wild-type C57BL/6NCrl mice (MGI ID 2683688) were obtained from Charles River Animal Facility by the Australian Phenomics Facility and were used unless otherwise stated. *MyD88*^*−/−*^ mice were a kind gift of Profs Narci Teoh and Geoff Farrell, ANU. *Tlr2*^*−/−*^ (Tlr2^tm1Aki^, MGI ID 2178675) and *Tlr4*^*−/−*^ mice (Tlr4^tm1Aki^, MGI ID 1860885), have been previously described^52^ and were compared to facility C57BL/6NCrl controls. All procedures were approved under protocols AE 2015/43, 2017/55, and 2018/55 by the Australian National University Animal Ethics and Experimentation Committee, in accordance with the National Health and Medical Research Council’s Australian Code for the Care and Use of Animals for Scientific Purposes and the ACT Animal Welfare Act 1992.

### Human samples

Experiments using human samples were conducted in accordance with human ethics protocols 2015/558 and 2016/071 as approved by the Australian National University Human Research Ethics Committee. Samples were collected after informed consent was obtained. Samples were collected from both male and female donors aged 20–35 years.

### In vitro NET generation

In vitro NETs from human CD57BL/6NCrl whole Blood Neutrophil Isolation were generated as described previously^27^. Briefly, neutrophils were isolated from human blood using the MACSxpress Whole Blood Neutrophil Isolation Kit-Human (Miltenyi Biotec) as per the manufacturer’s instructions. Neutrophils were plated at 1.5 × 10^6^ cells/well in 12-well plates (Costar/Corning) in 500 µL/well in MCDB 131/0.5% HIFBS. 50 nM PMA (Sigma) was added to plated neutrophils before incubation for 4 h at 37 °C, 5% CO_2_ to induce NET formation. Supernatants were carefully collected and the remaining neutrophil/NETs were gently washed twice with 1 mL PBS to remove all remaining PMA. To detach NETs from cell debris, NETs were digested in the well with ALU1 (NEB) at 4 U/mL in 400 mL/well in MCDB 131/0.5% HIFBS for 20 min at 37 °C. Digested NETs were collected by mixing vigorously and subsequent centrifugation at 300×*g* for 5 min at 4 °C to separate out remaining cell debris. Cell-free supernatant (soluble NETs) was collected into fresh tubes and stored at −20 °C until use.

### Flow cytometry

The following dyes were used for flow cytometry analysis: 7AAD (Life Technologies, Carlsbad, CA, USA Cat# A1310), Annexin V (BD Biosciences, Franklin Lakes, NJ, USA, Cat# 556419, RRID:AB_2665412), Live Dead Fixable Viability Dye (eBioscience, San Diego, CA, USA, Cat# 65-0865-14) DAPI (BioLegend, San Diego, CA, USA, Cat# 422801), Zombie Aqua (BioLegend Cat# 423102).

Conjugated antibodies against the following antigens were used for murine samples: CD25 (ThermoFisher Scientific, Waltham, MA, USA, Cat# 12-0251-82, RRID:AB_465607), CD3 (BioLegend Cat# 100335, RRID:AB_10898314), CD3 (BioLegend Cat# 100218, RRID:AB_1595492), CD4 (BD Biosciences Cat# 553051, RRID:AB_398528), CD4 (BioLegend Cat# 100414, RRID:AB_312699), CD44 (BioLegend Cat# 103020, RRID:AB_493683), CD44 (BioLegend Cat# 103047, RRID:AB_2562451), CD44 (BD Biosciences Cat# 553134, RRID:AB_394649), CD45.1 (BD Biosciences Cat# 612811, RRID:AB_2870136), CD45.2 (BD Biosciences Cat# 563051, RRID:AB_2737974), CD62L (BioLegend Cat# 104428, RRID:AB_830799), CD69 (BioLegend Cat# 104506, RRID:AB_313109), CD8 (BD Biosciences Cat# 565968, RRID:AB_2739421), FOXP3 (ThermoFisher Scientific Cat# 12-5773-80, RRID:AB_465935), RORγt (BD Biosciences Cat# 562894, RRID:AB_2687545), GM-CSF (BioLegend Cat# 505406, RRID:AB_315382), IFNγ (ThermoFisher Scientific Cat# 25-7311-82, RRID:AB_469680), IL-17a (BioLegend Cat# 506908, RRID:AB_536010), IL-17 (ThermoFisher Scientific Cat# 17-7177-81, RRID:AB_763580), pSTAT3 (pY705) (BD Biosciences Cat# 612569, RRID:AB_399860), pSTAT6 (pY641) (ThermoFisher Scientific Cat# 17-9013-42, RRID:AB_2573274), Conjugated antibodies against the following antigens were used for human samples: CD3 (BioLegend Cat# 317340, RRID:AB_2563408), CD4 (BioLegend Cat# 317432, RRID:AB_2028494), CD45RA (BioLegend Cat# 304128, RRID:AB_10708880), IFNγ (BioLegend Cat# 502515, RRID:AB_493029), IL-17a (BioLegend Cat# 512306, RRID:AB_961394). Flow cytometry was performed using a LSRII machine (BD Biosciences) and analyzed using FlowJo 10.5.0 software (BD Biosciences). In vitro experiments were gated on live, single cell, lymphocytes. Imaging flow cytometry was performed using the Amnis Imagestream (Luminex Corporation, Austin, TX, USA) at ×60 magnification and analyzed using IDEAS (version 6.2). Cells were analyzed after exclusion of out of focus, non-single cell events.

### Murine cell sorting and differentiation

Naive CD4^+^ T cells were isolated from spleens and lymph nodes of mice by fluorescence-activated cell sorting (FACS). In short, naive cells were isolated using a combination of the CD4 (L3T4) isolation kit (Miltenyi, Bergisch Gladbach, Germany, Cat# 130-117-043) and subsequent FACS sorting of viable (7AAD^−^) naive T cells (CD4^+^CD62L^+^CD44^−^) using the BD FACS Aria II (BD Biosciences). The purity of naive CD4^+^ cells was verified to be above 99% in all cases. In vitro murine T cell differentiation of Th1 and Th17 cells was performed as described previously^53^ using 1 μg/mL αCD3 (BioXcell Cat# BE0001-1) and 2 μg/mL αCD28 (BioXcell Cat# BE0015-1)with 200,000 naive T cells plated per well onto flat bottomed 96 well tissue culture plates (Corning). Regulatory T cells were differentiated by the addition of 2 ng/mL TGF-β (Peprotech) in the presence of 2.5 μg/mL αIFNγ and 2.5 μg/mL αIL-4 (BioXcell Cat # BE0055 and BE0045). Pathogenic Th17 cells were generated by addition of 30 ng/mL IL-6 (Peprotech), 20 ng/mL IL-23 and 100 μg/mL IL-1β (R&D Systems, MN, USA) ±2 ng/mL TGF-β with 2.5 μg/mL αIFNγ and 2.5 μg/mL αIL-4 and cultured for 3 days on anti-CD3 followed by 2 days of culture on uncoated plates.

### Histone killing assays

Sorted naive T cells from mice in Iscove’s Modified Dulbecco’s Medium (IMDM, ThermoFisher Cat# 12440053) +10% FCS were incubated with various concentrations of unfractionated whole histones from calf thymus (Sigma, Burlington, MA, USA Cat# H9250) or sterile water for 30 min to 4 h at 37 °C, 5% CO_2_. Cell death was determined either by 7AAD positivity or using 7AAD in conjunction with Annexin V FITC in Annexin V binding buffer (BioLegend Cat# 422201).

### Human cell sorting

Blood from healthy controls was collected into sodium heparin vacutainers. Peripheral blood mononuclear cells (PBMCs) were then isolated by gradient centrifugation using Lymphoprep™ (Stemcell Technologies, Vancouver, Canada, Cat# 07801) using the method described by the manufacturer. Isolated PBMCs were stained for surface expression of CD3 (Af488, HIT3a), CD4 (PerCP, OKT4), and CD45RA (APC-Cy7, HI100). Viable (DAPI^−^), naive T cells (CD3^+^CD4^+^CD45RA^+^) were sorted by FACS using either the BD FACS Aria II (BD Biosciences) or the FACS Melody (BD Biosciences). The purity of naive CD4^+^ cells was verified to be above 99% in all cases.

### Human T cell differentiation

Isolated human naive T cells were stimulated for 6 days in 96 well, flat bottomed plates (Corning) using 1 µg/mL of plate-bound anti-CD3 (BioLegend Cat# 317302, RRID:AB_571927) and 2 µg/mL anti-CD28 (BioLegend Cat# 302902, RRID:AB_314304). Cells were seeded at 100,000 naive T cells per well. Th1 cells were generated by addition of 10 ng/mL hIFNγ, 10 ng/mL IL-12 and 100 U/mL hIL-2 (all from Miltenyi). Th17 cells were generated using 10 ng/mL hIL-1β, 10 ng/mL hIL-6, 10 ng/mL hIL-23, 100 U/mL hIL-2, 10 ng/mL TGF-β (all from Miltenyi) with 10 µg/mL anti-IFNγ (BioLegend Cat# 502402, RRID:AB_315223). Cells were incubated at 37 °C, 5% CO_2_. Cells were cultured in complete IMDM (ThermoFisher, with 10% FCS, Penicillin, streptomycin and glutamine).

### Addition of histones, NETs, and other stimulants to T cell differentiation cultures

Histones (0–20 μg/mL, Sigma Cat# H9250), PAM3CSK4 (1 μg/mL, Invivogen, San Diego, CA, USA Cat# tlrl-pms), PAM2CSK4 (1 μg/mL, Invivogen, Cat# tlrl-pm2s-1), PHAD (1 μg/mL, Avanti Polar Lipids, Alabaster, AL, USA, Cat 699800) or CpG ODN2395 (1 μg/mL, Miltenyi Cat# 130-100-282) diluted in complete IMDM were added to naive T cells at the time of culture. For stimulation with NETs, NET preparations in MCDB 131 media or MCDB 131 media alone were similarly added at the start of the culture period. For experiments inhibiting histones/NETs, the inhibitor β-methyl-cellobioside sulfate (mCBS) (100 μg/mL) or PBS was added simultaneously with the addition of histones or NETs to cultures. Unless otherwise stated, in all experiments, histones/TLR agonists/NETs/mCBS were added at the start of the culture period and remained in the media for the duration of the cell culture. TLR4 activity of PHAD was confirmed using HEK-Blue™ mTLR4 Cells (Invivogen, Cat# hkb-mtlr4) as described by the manufacturer.

### Histone/T cell co-stimulation assays

Sorted naive T cells were stimulated with 1 µg/mL anti-CD3 in the presence or absence of 2 µg/mL anti-CD28. Known concentrations of calf thymus histone or sterile water were then added to the cells. Cells were incubated for 30 min–24 h (as described) at 37 °C, 5% CO_2_. Surface staining was subsequently performed.

### Proliferation

For proliferation analysis isolated naive T cells were labeled with CTV (ThermoFisher Scientific Cat# C34571) as previously described^54^. The cell division index was calculated using the formula (Eq. 1):

1 $${{{{{\rm{Division}}}}}}\,{{{{{\rm{index}}}}}}={{{{{\rm{sum}}}}}}({{i}}* {{N}}({{i}})/{2}^{{{i}}})/{{{{{\rm{sum}}}}}}({{N}}({{i}})/{2}^{{{i}}})$$

where *i* = division number (undivided = 0) and *N*(*i*) = number of events in division *i*.

### Intracellular staining of cytokines and transcription factors

Intracellular cytokine staining was performed after re-stimulation of cells with 50 nM PMA (Sigma Aldrich Cat# P8139) and 500 ng/mL Ionomycin (Sigma Cat# I3909) in the presence of Golgi stop (BD Biosciences Cat# 654724) for 4.5 h as described previously^53^.

### Analysis of phosphorylation by flow cytometry (Phosflow)

Sorted cells were rested for 2 h on ice in PBS before stimulation in Aim V serum-free media (Gibco, ThermoFisher Scientific, Cat# 12055083). Cells were then washed with PBS, fixed using 1.5% formaldehyde, and permeabilized using methanol as previously described^55^.

### qPCR analysis

Cell pellets from d5/6 of cell culture were resuspended in RNA Tri-Flüssig (Bio&SELL, Feucht, Germany). RNA was isolated using the method described by Invitrogen™ for TRIzol™ Reagent. RNA was quantified by absorbance using a SpectraMax® QuickDrop Micro-Volume Spectrophotometer (Molecular Devices, CA, USA). cDNA was synthesized using RevertAid Reverse Transcriptase (ThermoFisher Scientific). qPCR was performed using 5× Hot Start Taq EvaGreen® qPCR mix (no ROX) (Axon Labortechnik, Kaiserslautern, Germany) as per the manufacturer’s instructions in combination with the StepOnePlus™ Real-Time PCR System (Applied Biosystems, CA, USA). Fold change in gene expression was determined by calculation of 2^−2ΔΔCt^ with *Hprt* expression used as a housekeeping control. Raw ΔΔ*Ct* values were used for the calculation of statistical significance.

### Oligonucleotides

The following primer pairs were used for analysis of gene expression by qPCR. *Hprt*, 5′-GTTGGATACAGGCCAGACTTTGT-3′ (forward) and 5′-GAGGGTAGGCTGGCCTATAGGCT-3′ (reverse)(Sigma); *Irf4*, 5′-GCAGCTCACTTTGGATGC A-3′ (forward) and 5′-TGGCATCAAGTTTCA CAAA-3′ (reverse) (Metabion, Planegg, Germany); *Ahr*, 5′-CTGGTTGTCAATGCCT (forward) and 5′-CGGTCTTCTGGAGCTC-3′ (reverse); *Ccr6*, 5′-CCTCACATTCTGGAGC-3′ (forward) and 5′-CCTCACATTCTGGAGC-3′ (reverse); *Il-17a*, 5′-TTTAACTCCCGCAAAA-3′ (forward) and 5′-CTTTCCCTCCTGACAC-3′ (reverse); *Il-23r*, 5′-GCCAAGAAGATCCCGA-3′ (forward) and 5′-TCAGTGCTACAGGACA-3′ (reverse); *Hif1a*, 5′-ATAGCTTCGCCTCAGA-3′ (forward) and 5′-CAGTCACCTGCTGCAA-3′ (reverse); *Tgfbr3*, 5′-TCTCCGCTGAGTGGTA-3′ (forward) and 5′-CCGACTCCAAGTAGCC-3′ (reverse) (all from Biomers.net, Ulm, Germany)

### In vivo histone delivery

Eight-week-old C57BL/6NCrl mice were injected intravenously with 1 μg/g histone diluted in PBS, or PBS only. 48 h later, blood, spleens, mesenteric lymph nodes, and Peyer’s patches were collected for analysis. For experiments where TCR stimulation was also required, 24 h prior to histone delivery, mice were injected intravenously with 0.1 μg/g anti-CD3.

### Adoptive T cell transfer and in vivo histone delivery

CD4^+^ T cells were isolated from spleens and lymph nodes of CD45.2 *Tlr2*^*+/+*^ and *Tlr2*^−/−^ mice by MACS using the CD4 (L3T4) isolation kit (Miltenyi) and 6 × 10^6^ cells injected intravenously into 8-week-old B6NCrl-Ptprc(a)/Anu mice. Fort-eight hours post-adoptive transfer, in vivo histone delivery (with TCR stimulation) was performed as described above.

### Multiplex ELISA

The concentration of cytokines in plasma from cardiac blood following in vivo histone delivery was determined using the MILLIPLEX MAP mouse cytokine/chemokine multiplex assay (Merck Millipore, Darmstadt, Germany) as described by the manufacturer.

### Experimental design and data normalization

For in vivo experiments, treatments were randomized within and across cages of mice. Researchers were blinded to the treatment each animal received until after data were analyzed. Similarly, researchers were blinded to the genotype of animals in in vitro experiments comparing knockout and wild-type animals until after data were analyzed. Where experiments involved treatment of cells in vitro with histones/NETs/mCBS, samples were compared within one donor mouse treated vs untreated, indicated numbers represent the total number of mice used in each experiment. To allow for the combination of data across experiments, some data were normalized. In these cases, data were normalized to the mean of the untreated group of mice of the same genotype in each experiment to conserve equal variance.

### Statistical analysis

For all cases, data were presented as the mean ± the standard error of the mean (SEM). Data were analyzed using paired and unpaired t-tests, one-way ANOVA, two-way ANOVA or mixed-effects model (restricted maximum likelihood; REML) with corrections for multiple comparisons, as stated in Figure legends. Where in vitro experiments compared treatment effects, individual donor mice were used as a blocking factor. In all cases, a normal distribution of data was assumed, a two-tailed test applied and a confidence interval of 95% was used with adjusted *p* values of *p* < 0.05 considered significant. Statistical data analysis was performed using GraphPad Prism (version 8.2.1) software.

### Reporting summary

Further information on research design is available in the Nature Research Reporting Summary linked to this article.

## Supplementary information

Supplementary Information

Reporting Summary

## Data Availability

All source data are provided with this paper and available upon reasonable request to the corresponding author. Source data are provided with this paper.

## Source data

Source data

## Materials availability

The compound, mCBS, has been produced by Sirtex Medical Ltd and is available to researchers free of charge provided an MTA is in place.

## References

[1] Brinkmann V (2004). Neutrophil extracellular traps kill bacteria. Science.

[2] Kaplan MJ, Radic M (2012). Neutrophil extracellular traps: double-edged swords of innate immunity. J. Immunol..

[3] Khandpur R (2013). NETs are a source of citrullinated autoantigens and stimulate inflammatory responses in rheumatoid arthritis. Sci. Transl. Med..

[4] Urban CF (2009). Neutrophil extracellular traps contain calprotectin, a cytosolic protein complex involved in host defense against *Candida albicans*. PLoS Pathog..

[5] Marsman G, Zeerleder S, Luken BM (2016). Extracellular histones, cell-free DNA, or nucleosomes: differences in immunostimulation. Cell Death Dis..

[6] Abrams ST (2013). Circulating histones are mediators of trauma-associated lung injury. Am. J. Respir. Cell Mol. Biol..

[7] Kutcher, M. E. et al. Extracellular histone release in response to traumatic injury: implications for a compensatory role of activated protein C. *J. Trauma Acute Care Surg.* **73**, 1389–1394 (2012). 10.1097/TA.0b013e318270d595 PMC3577065 23188230

[8] Xu J (2009). Extracellular histones are major mediators of death in sepsis. Nat. Med..

[9] Tsourouktsoglou T-D (2020). Histones, DNA, and citrullination promote neutrophil extracellular trap inflammation by regulating the localization and activation of TLR4. Cell Rep..

[10] Borissoff JI (2013). Elevated levels of circulating DNA and chromatin are independently associated with severe coronary atherosclerosis and a prothrombotic state significance. Arter. Thromb. Vasc. Biol..

[11] Monach, P. A. et al. A broad screen for targets of immune complexes decorating arthritic joints highlights deposition of nucleosomes in rheumatoid arthritis. *Proc. Natl Acad. Sci. USA*. **106**, 15867–15872 (2009). 10.1073/pnas.0908032106 PMC2747210 19720992

[12] Ouyang W, Kolls JK, Zheng Y (2008). The biological functions of T helper 17 cell effector cytokines in inflammation. Immunity.

[13] Ma CS (2008). Deficiency of Th17 cells in hyper IgE syndrome due to mutations in STAT3. J. Exp. Med..

[14] Bettelli E (2006). Reciprocal developmental pathways for the generation of pathogenic effector TH17 and regulatory T cells. Nature.

[15] Cua DJ (2003). Interleukin-23 rather than interleukin-12 is the critical cytokine for autoimmune inflammation of the brain. Nature.

[16] Chung Y (2009). Critical regulation of early Th17 cell differentiation by interleukin-1 signaling. Immunity.

[17] Ivanov II (2006). The orphan nuclear receptor RORγt directs the differentiation program of proinflammatory IL-17+ T helper cells. Cell.

[18] Sandquist, I. & Kolls, J. Update on regulation and effector functions of Th17 cells. *F1000Res* **7**, 205 (2018). 10.12688/f1000research.13020.1 PMC5820607 29527301

[19] Matusevicius D (1999). Interleukin-17 mRNA expression in blood and CSF mononuclear cells is augmented in multiple sclerosis. Mult. Scler..

[20] Tzartos JS (2008). Interleukin-17 production in central nervous system-infiltrating T cells and glial cells is associated with active disease in multiple sclerosis. Am. J. Pathol..

[21] Chabaud M, Fossiez F, Taupin J-L, Miossec P (1998). Enhancing effect of IL-17 on IL-1-induced IL-6 and leukemia inhibitory factor production by rheumatoid arthritis synoviocytes and its regulation by Th2 cytokines. J. Immunol..

[22] Kirkham BW (2006). Synovial membrane cytokine expression is predictive of joint damage progression in rheumatoid arthritis: a two‐year prospective study (the DAMAGE study cohort). Arthritis Rheum..

[23] Abi Abdallah DS, Egan CE, Butcher BA, Denkers EY (2011). Mouse neutrophils are professional antigen-presenting cells programmed to instruct Th1 and Th17 T-cell differentiation. Int. Immunol..

[24] Tecchio C, Micheletti A, Cassatella MA (2014). Neutrophil-derived cytokines: facts beyond expression. Front. Immunol..

[25] Warnatsch A, Ioannou M, Wang Q, Papayannopoulos V (2015). Neutrophil extracellular traps license macrophages for cytokine production in atherosclerosis. Science.

[26] Lambert S (2019). Neutrophil extracellular traps induce human Th17 cells: effect of psoriasis-associated TRAF3IP2 genotype. J. Invest. Dermatol..

[27] O’Meara CH (2020). Neutralizing the pathological effects of extracellular histones with small polyanions. Nat. Commun..

[28] Brustle A (2007). The development of inflammatory T(H)-17 cells requires interferon-regulatory factor 4. Nat. Immunol..

[29] Mudter J (2011). IRF4 regulates IL-17A promoter activity and controls RORgammat-dependent Th17 colitis in vivo. Inflamm. Bowel Dis..

[30] Semeraro F (2011). Extracellular histones promote thrombin generation through platelet-dependent mechanisms: involvement of platelet TLR2 and TLR4. Blood.

[31] Allam R (2012). Histones from dying renal cells aggravate kidney injury via TLR2 and TLR4. J. Am. Soc. Nephrol..

[32] Roach JC (2005). The evolution of vertebrate Toll-like receptors. Proc. Natl Acad. Sci. USA.

[33] Reynolds JM, Dong C (2013). Toll-like receptor regulation of effector T lymphocyte function. Trends Immunol..

[34] Kawasaki T, Kawai T (2014). Toll-like receptor signaling pathways. Front. Immunol..

[35] Harris TJ (2007). Cutting edge: an in vivo requirement for STAT3 signaling in TH17 development and TH17-dependent autoimmunity. J. Immunol..

[36] Nakahara M (2013). Recombinant thrombomodulin protects mice against histone-induced lethal thromboembolism. PLoS ONE.

[37] Silk E, Zhao H, Weng H, Ma D (2017). The role of extracellular histone in organ injury. Cell Death Dis..

[38] Ekaney ML (2014). Impact of plasma histones in human sepsis and their contribution to cellular injury and inflammation. Crit. Care.

[39] Gauthier VJ, Tyler LN, Mannik M (1996). Blood clearance kinetics and liver uptake of mononucleosomes in mice. J. Immunol..

[40] Hu Z (2017). Neutrophil extracellular traps induce IL-1β production by macrophages in combination with lipopolysaccharide. Int. J. Mol. Med..

[41] Minns D (2021). The neutrophil antimicrobial peptide cathelicidin promotes Th17 differentiation. Nat. Commun..

[42] Tohme, S. et al. Computational analysis supports IL-17A as a central driver of neutrophil extracellular trap–mediated injury in liver ischemia reperfusion. *J. Immunol*. **202**, 268–277 (2018). 10.4049/jimmunol.1800454 PMC6310076 30504418

[43] Zuo, Y. et al. Neutrophil extracellular traps in COVID-19. *JCI Insight* **5**, e138999 (2020). 10.1172/jci.insight.138999 PMC7308057 32329756

[44] Lam FW, Cruz MA, Parikh K, Rumbaut RE (2016). Histones stimulate von Willebrand factor release in vitro and in vivo. Haematologica.

[45] Hakkim A (2010). Impairment of neutrophil extracellular trap degradation is associated with lupus nephritis. Proc. Natl Acad. Sci. USA.

[46] Holdenrieder S (2001). Nucleosomes in serum of patients with benign and malignant diseases. Int. J. Cancer.

[47] Yang, X. et al. Clinical course and outcomes of critically ill patients with SARS-CoV-2 pneumonia in Wuhan, China: a single-centered, retrospective, observational study. *Lancet Respir. Med.* (2020). 10.1016/S2213-2600(20)30079-5 PMC7102538 32105632

[48] Martin JC, Baeten DL, Josien R (2014). Emerging role of IL-17 and Th17 cells in systemic lupus erythematosus. Clin. Immunol..

[49] Charles KA (2009). The tumor-promoting actions of TNF-α involve TNFR1 and IL-17 in ovarian cancer in mice and humans. J. Clin. Invest..

[50] Derhovanessian E (2009). Pretreatment frequency of circulating IL‐17+ CD4+ T‐cells, but not Tregs, correlates with clinical response to whole‐cell vaccination in prostate cancer patients. Int. J. Cancer.

[51] Kuang D (2010). Activated monocytes in peritumoral stroma of hepatocellular carcinoma promote expansion of memory T helper 17 cells. Hepatology.

[52] Blumenthal A (2009). RP105 facilitates macrophage activation by *Mycobacterium tuberculosis* lipoproteins. Cell Host Microbe.

[53] Wilson AS (2019). Protection from EAE in DOCK8 mutant mice occurs despite increased Th17 cell frequencies in the periphery. Eur. J. Immunol..

[54] Quah BJ, Parish CR (2012). New and improved methods for measuring lymphocyte proliferation in vitro and in vivo using CFSE-like fluorescent dyes. J. Immunol. Methods.

[55] Krutzik PO, Nolan GP (2003). Intracellular phospho-protein staining techniques for flow cytometry: monitoring single cell signaling events. Cytom. A.

