# Supplementary material for: Neutrophil extracellular traps and their histones promote Th17 cell differentiation directly via TLR2
Source: Nat Commun. 2022 Jan 26;13:528. doi: 10.1038/s41467-022-28172-4 (PMC8792063; doi:10.1038/s41467-022-28172-4)
Supplement: Supplementary file 1 — Supplementary Information [file 41467_2022_28172_MOESM1_ESM.pdf]

Supplementary Information

Neutrophil extracellular traps and their histones promote Th17 cell differentiation directly via TLR2

Alicia S. Wilson[1,2], Katrina L. Randall[1,3], Jessica A. Pettitt[1], Julia Ellyard[1], Antje Blumenthal[4], Anselm Enders[1], Benjamin J. Quah[1], Tobias Bopp[2], Christopher R Parish[1], Anne Brüstle[1]*

[1]The John Curtin School of Medical Research, The Australian National University, Canberra, ACT, Australia.

[2] Institute for Immunology, University Medical Center, Johannes Gutenberg University Mainz, Mainz, Germany

[3]ANU Medical School, The Australian National University, Canberra, ACT, Australia.

[4]The University of Queensland Diamantina Institute, The University of Queensland, Brisbane, QLD, Australia

* Correspondence to: anne.bruestle@anu.edu.au

Contents

Supplementary Figure 1

Supplementary Figure 2

Supplementary Figure 3

Supplementary Figure 4

Supplementary Figure 5

Supplementary Figure 6

Supplementary Figure 7

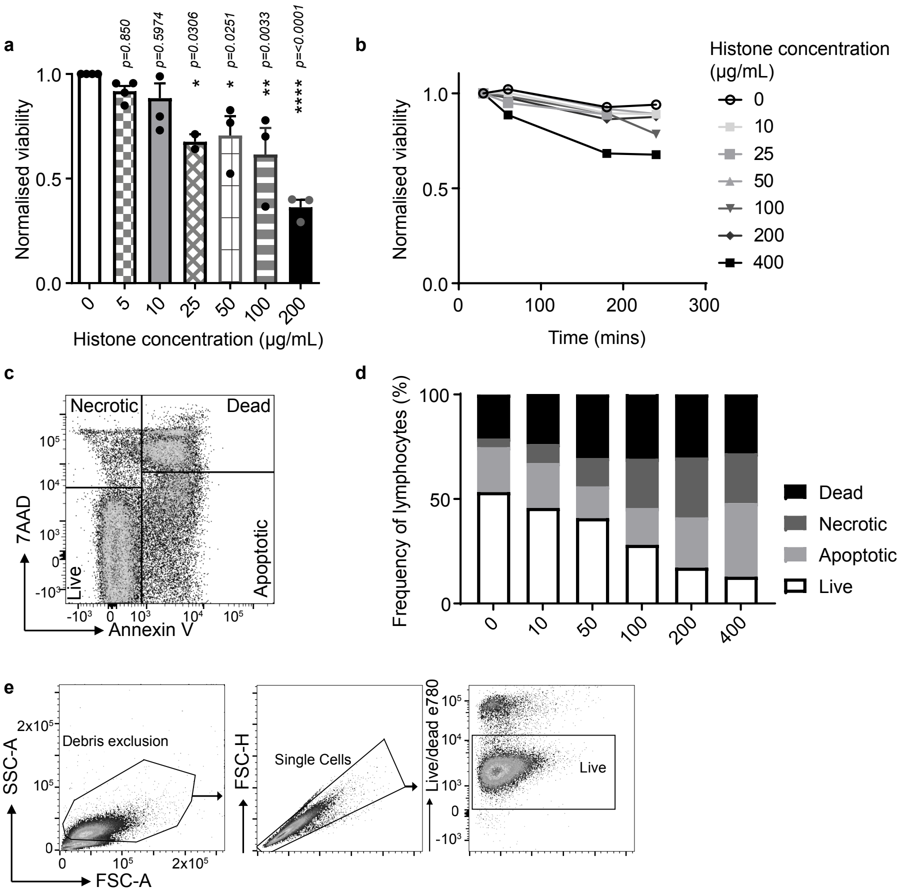

Supplementary Figure 1: **CD4 T cells are susceptible to histone-induced cell death.** Normalized cell viability (frequency of 7AAD negative cells compared to 0 μg/mL histone control) of sorted CD4 T cells after incubation with increasing concentrations of histones at **a** 30 minutes and **b** up to 4 hours (240 min). **c** Gating to define necrotic, apoptotic and dead cells by 7AAD and Annexin V staining was used to define **d** death responses to increasing doses of histone. **e** Gating strategy for defining live cells after *in vitro* culture. Bars show mean ± SEM. 'ns' (not significant), $p>0.05$; '*', $p<0.05$; '**', $p<0.01$; '****', $p<0.001$. Where significance is not indicated, differences were ns. Statistical significance was determined using a one-way ANOVA with Dunnett's multiple comparisons test. Data points represent individual mice. All data are representative of a minimum of two independent experiments. Source data are provided as a Source Data file.

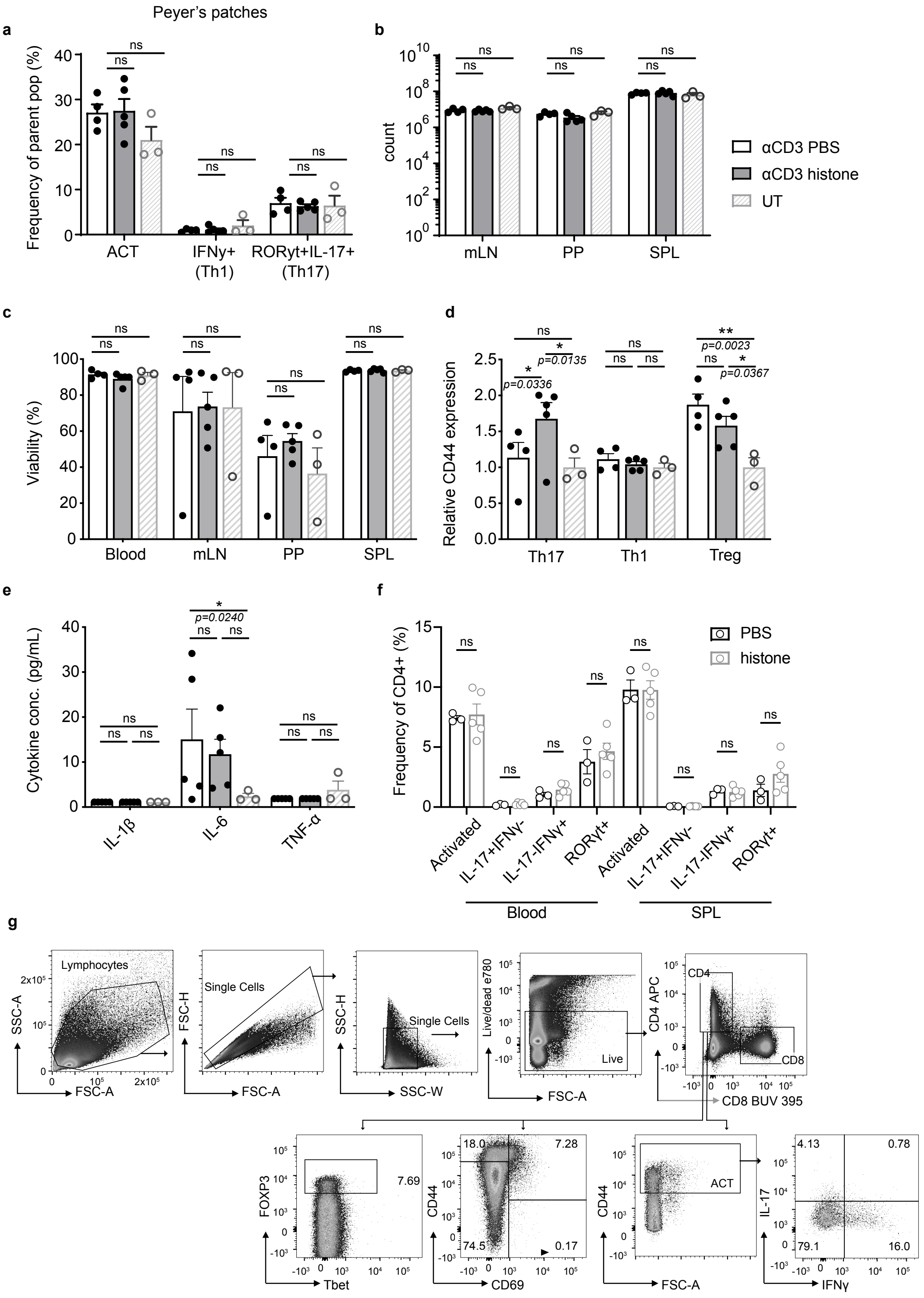

a
Peyer's patches
Frequency of parent pop (%)
ACT
IFNy+ (Th1)
RORyt+IL-17+ (Th17)
b
count
mLN
PP
SPL
αCD3 PBS
αCD3 histone
UT
c
Viability (%)
Blood
mLN
PP
SPL
d
Relative CD44 expression
p=0.0336
p=0.0135
p=0.0023
p=0.0367
Th17
Th1
Treg
e
Cytokine conc. (pg/mL)
p=0.0240
IL-1β
IL-6
TNF-α
f
Frequency of CD4+ (%)
PBS
histone
Activated
IL-17+IFNγ-
IL-17-IFNγ+
RORγt+
Blood
SPL
g
Lymphocytes
Single Cells
Live
CD4
CD8
ACT
SSC-A
FSC-A
FSC-H
SSC-H
SSC-W
Live/dead e780
CD4 APC
CD8 BUV 395
FOXP3
Tbet
CD44
CD69
IL-17
IFNγ

Supplementary Figure 2: **Intravenous histone delivery does not impact T cell activation or differentiation in the Peyer's Patches or total cell viability in several lymphoid organs. a** Activated (ACT; $CD44^+$) T cells, Th1 ($IFN\gamma^+$) and Th17 ($ROR\gamma t^+ IL\text{-}17^+$) cells in Peyer's Patches are displayed as a frequency of live CD4 T cells after delivery of αCD3 mAb with or without histones. **b** Number and **c** frequency of viable cells in the blood (c only), mesenteric lymph nodes (mLN), Peyer's Patches (PP) and spleens (SPL) of mice after delivery of αCD3 with or without histones. **d** Relative CD44 expression (MFI normalised to untreated control (UT)) of Th17, Th1 and Treg cells and **e** concentration of circulating cytokines IL-1β, IL-6 and TNF-α from plasma 2 days after delivery of αCD3 mAb with or without histones. **f** Activated, Th1, Th17 and RORγt cell populations as a percentage of $CD4^+$ T cells from the blood and spleens of mice after delivery with PBS (n=3) or histones alone (n=5). **g** Exemplary FACS plots and gating strategy for (a-d,f). Data points represent individual mice, bars show mean ± SEM. 'ns' (not significant), $p>0.05$; '*', $p<0.05$; '**', $p<0.01$. Statistical significance was determined using a two-way ANOVA with Tukey's multiple comparisons test. Source data are provided as a Source Data file.

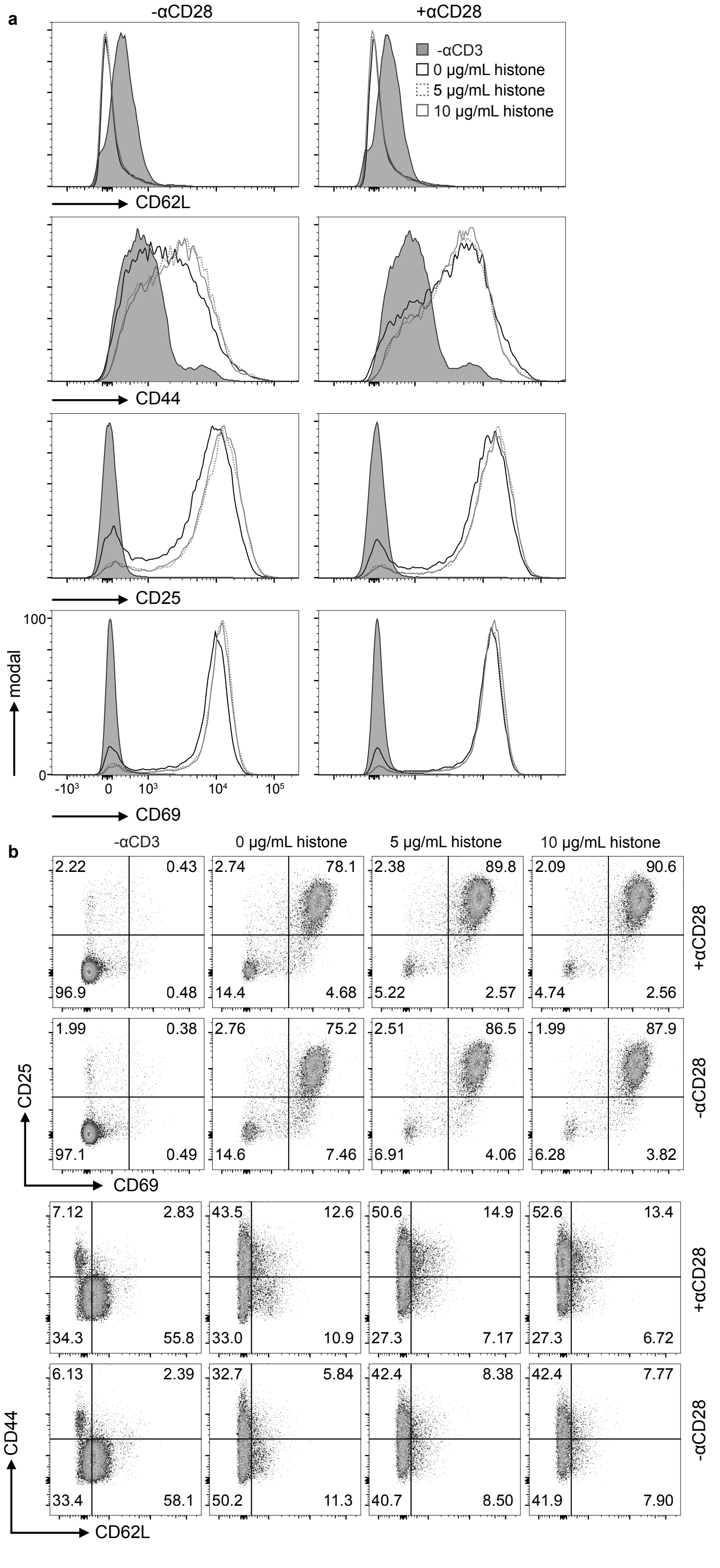

Supplementary Figure 3: Histones promote TCR dependent naïve CD4 T cell activation. a Example histograms and **b** flow cytometry plots showing expression of T cell activation markers CD62L (naïve T cell marker), CD25, CD69 and CD44 after 16 hours co-culture with 1μg/mL αCD3 and 0-10 μg/mL histone in the presence or absence (+/-) of αCD28 measured by flow cytometry. Figures representative of 5 mice per group.

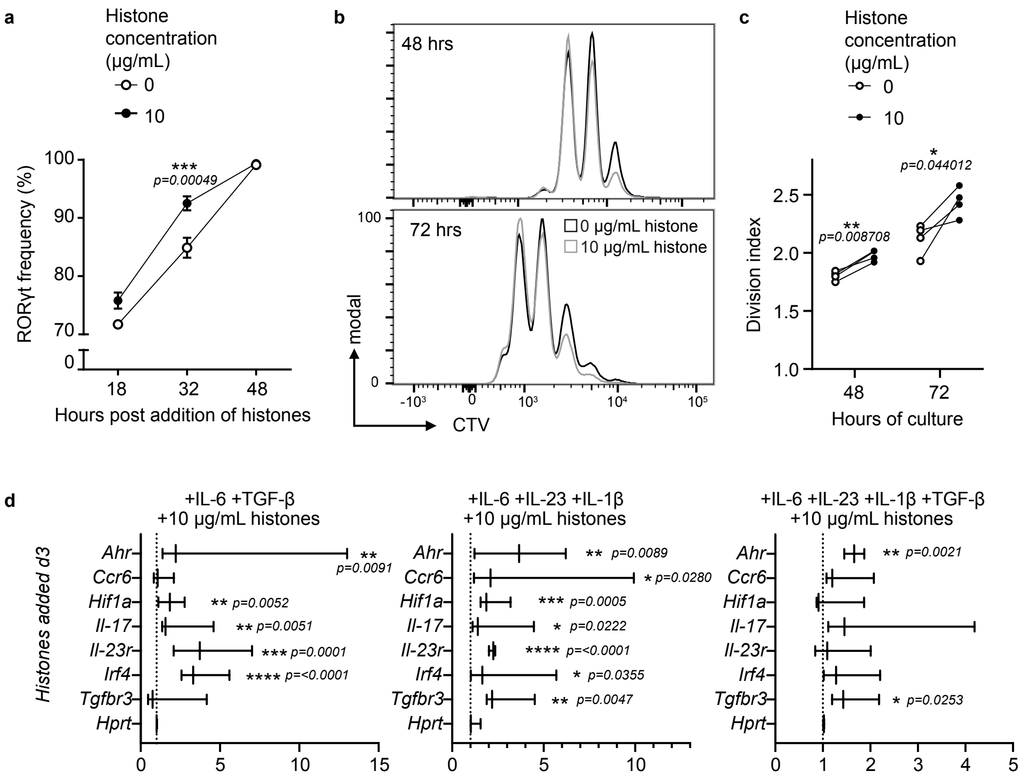

Supplementary Figure 4: **Addition of histones has a small impact upon murine Th17 cell proliferation and promotes the expression of Th17 cell genes. a** Expression of RORγt by Th17 cells in culture over time in the presence or absence of histones measured by flow cytometry n=3. **b** Proliferation of murine Th17 cell cultures in the presence or absence of histones was measured by dilution of CTV using flow cytometry and **c** division index calculated using equation 1 after 48 and 72 hours of culture n=3. **d** Relative gene expression (fold change) of non-pathogenic (left) and pathogenic Th17 cells +/- TGF-β treated with histones after 3 days of differentiation compared to H_2O treated controls. Gene expression was measured on day 5 of culture with *Hprt* expression was used as a housekeeping control n=3. Bars show (a,c) mean ± SEM or (d) range and distribution. 'ns' (not significant), p>0.05; '*', p<0.05; '**', p<0.01; '***', p<0.001; '****', p<0.0001. Where significance is not indicated, differences were ns. Statistical significance was determined using (a) two-way ANOVA with Šídák's multiple comparisons test (c) multiple two-tailed t tests with Holm-Sidak correction, (d) one sample, two-tailed T test (performed on ΔΔC*t* values). Source data are provided as a Source Data file.

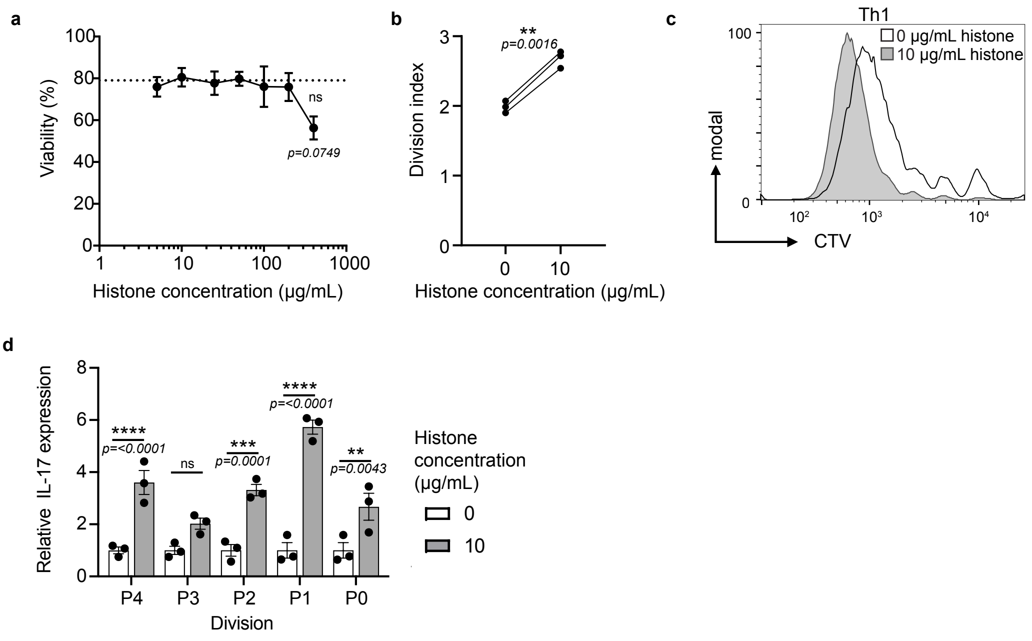

Supplementary Figure 5: **Low concentrations of histones are not toxic to human peripheral blood mononuclear cells and increase Th17 cell proliferation. a** Cell viability of isolated human PBMCs after incubation with increasing concentrations of histones for 4 hours with a dotted line indicating baseline cell viability with 0 µg/mL histone. Bars show mean ± SD. **b** Division index of human Th17 cells differentiated in the presence or absence of histones calculated using equation 1. **c** Representative histogram of CTV dilution by human cells in Th1 culture conditions in the presence or absence of histones measured by flow cytometry. **d** Relative IL-17 expression from each cell division (P0 *undivided* – P4) of human Th17 cells differentiated with or without histones. Bars show mean (a) ± SD or (d) ± SEM. 'ns' (not significant), $p>0.05$; '**', $p<0.01$; '***', $p<0.001$; '****', $p<0.0001$. Where significance is not indicated, differences were ns. Statistical significance was determined using (a) one-way ANOVA (comparing values to 0 µg/mL condition) with Dunnett's multiple comparisons test, (b) paired two-tailed T test or (d) two-way ANOVA with Šídák's multiple comparisons test. Data points represent individual donors. Source data are provided as a Source Data file.

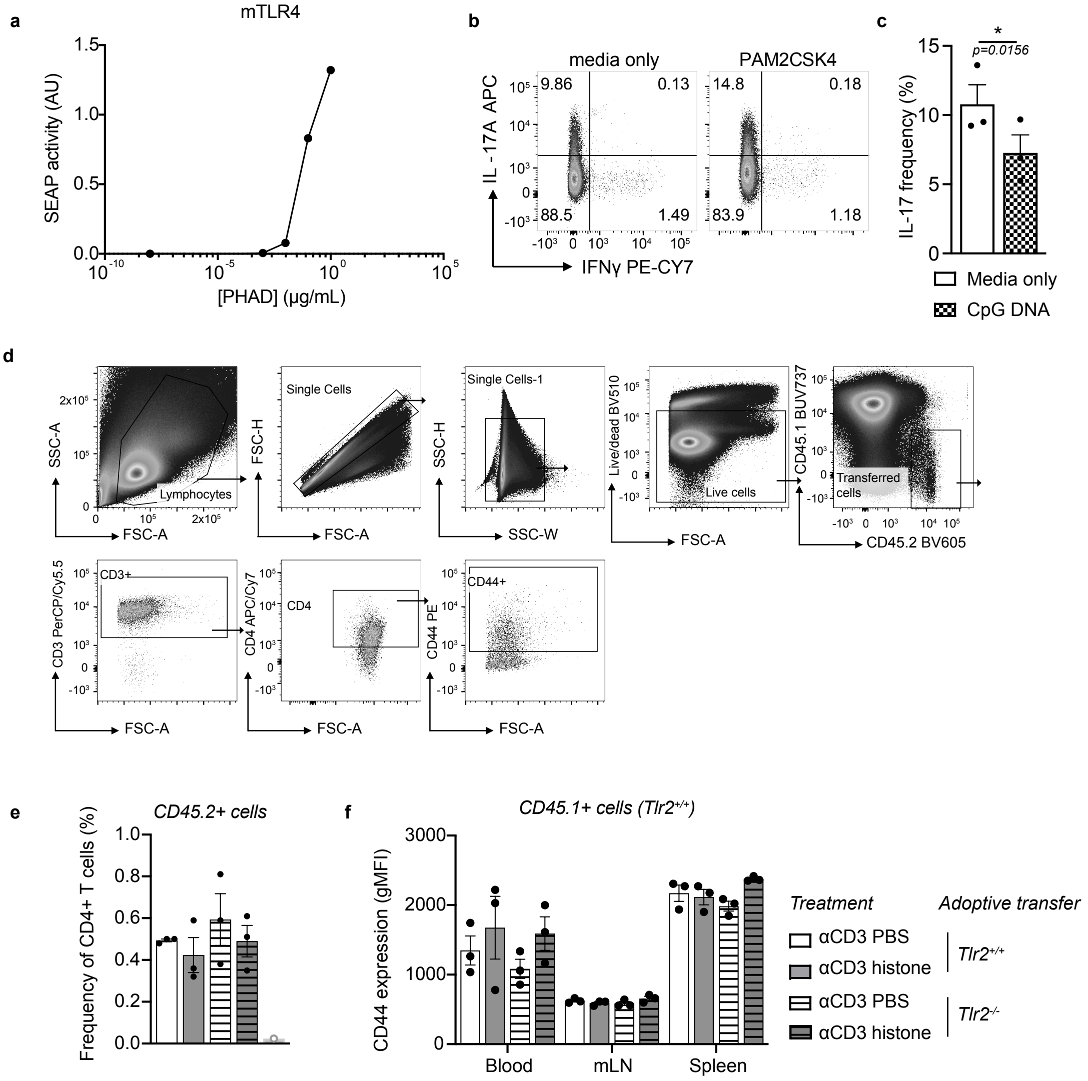

Supplementary Figure 6: **Histone-induced Th17 cell differentiation is dependent on TLR2. a** TLR4 stimulating activity of PHAD measured by secreted embryonic alkaline phosphatase (SEAP) activity of HEK-Blue™ mTLR4 cells. **b** Representative IL-17 and IFNγ expression measured by flow cytometry of Th17 cells differentiated with or without the addition of PAM2CSK4 **c** IL-17 expression measured by flow cytometry of Th17 cells differentiated in the presence or absence of CpG rich DNA n=3. **d** Exemplary FACS plots and gating strategy **e** frequency of transferred ($CD45.2^+$) cells from total $CD4^+$ T cells and **f** CD44 expression (gMFI) of $CD45.1^+$ $CD4^+$*Tlr2*$^{+/+}$ T cells from mice receiving CD4 T cells from *Tlr2*$^{+/+}$ and *Tlr2*$^{-/-}$ mice 48hrs before low dose αCD3 and 72h before intravenous histone injections n=3. . Where significance is not indicated, differences were ns, p>0.05; '*', p<0.05. Statistical significance was determined using (c) paired two-tailed T test, (e) one-way ANOVA or (f) two-way ANOVA with Tukey's multiple comparisons test. Source data are provided as a Source Data file.

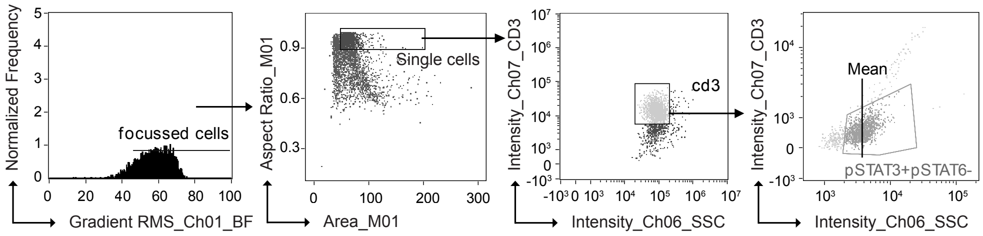

Supplementary Figure 7: **Imaging cytometry gating strategy**

Gating strategy for defining cells with mean fluorescence values for pSTAT3. Focussed, single cell, CD3$^+$ cells were gated and a population of pSTAT3 single positive cells defined. The geometric mean of this population was calculated and a 'mean' gate drawn including all cells with fluorescence intensities within 50 units of the geometric mean.
