# Supplementary material for: Neutrophil extracellular traps and their histones promote Th17 cell differentiation directly via TLR2
Source: Nat Commun. 2022 Jan 26;13:528. doi: 10.1038/s41467-022-28172-4 (PMC8792063; doi:10.1038/s41467-022-28172-4)
Supplement: Supplementary file 2 — Reporting Summary [file 41467_2022_28172_MOESM2_ESM.pdf]

nature research

Corresponding author(s): A/Prof Anne Bruestle

Last updated by author(s): Aug 27, 2021

Reporting Summary

Nature Research wishes to improve the reproducibility of the work that we publish. This form provides structure for consistency and transparency in reporting. For further information on Nature Research policies, see Authors & Referees and the Editorial Policy Checklist .

Statistics

For all statistical analyses, confirm that the following items are present in the figure legend, table legend, main text, or Methods section.

n/a	Confirmed	
[ ]	[x]	The exact sample size (*n*) for each experimental group/condition, given as a discrete number and unit of measurement
[ ]	[x]	A statement on whether measurements were taken from distinct samples or whether the same sample was measured repeatedly
[ ]	[x]	The statistical test(s) used AND whether they are one- or two-sided<br>*Only common tests should be described solely by name; describe more complex techniques in the Methods section.*
[ ]	[x]	A description of all covariates tested
[ ]	[x]	A description of any assumptions or corrections, such as tests of normality and adjustment for multiple comparisons
[ ]	[x]	A full description of the statistical parameters including central tendency (e.g. means) or other basic estimates (e.g. regression coefficient) AND variation (e.g. standard deviation) or associated estimates of uncertainty (e.g. confidence intervals)
[x]	[ ]	For null hypothesis testing, the test statistic (e.g. *F*, *t*, *r*) with confidence intervals, effect sizes, degrees of freedom and *P* value noted<br>*Give P values as exact values whenever suitable.*
[x]	[ ]	For Bayesian analysis, information on the choice of priors and Markov chain Monte Carlo settings
[x]	[ ]	For hierarchical and complex designs, identification of the appropriate level for tests and full reporting of outcomes
[x]	[ ]	Estimates of effect sizes (e.g. Cohen's *d*, Pearson's *r*), indicating how they were calculated

Our web collection on statistics for biologists contains articles on many of the points above.

Software and code

Policy information about availability of computer code

Data collection	Flowjo 10.5.0
Data analysis	Flow cytometry was performed using a LSRII machine (BD Biosciences) and analyzed using FlowJo 10.5.0 software (Tree Star). In vitro experiments were gated on live, single cell, lymphocytes. Imaging flow cytometry was performed using the Amnis Imagestream at 60X magnification and analyzed using IDEAS (version 6.2). Cells were analyzed after exclusion of out of focus, non-single cell events. Graphpad Prism version 8.2.1 was used to visualise statistical analysis

For manuscripts utilizing custom algorithms or software that are central to the research but not yet described in published literature, software must be made available to editors/reviewers. We strongly encourage code deposition in a community repository (e.g. GitHub). See the Nature Research guidelines for submitting code & software for further information.

Data

Policy information about availability of data

All manuscripts must include a data availability statement. This statement should provide the following information, where applicable:

- Accession codes, unique identifiers, or web links for publicly available datasets
- A list of figures that have associated raw data
- A description of any restrictions on data availability

Data sharing not applicable to this article as no datasets were generated or analyzed during the current study.

Field-specific reporting

Please select the one below that is the best fit for your research. If you are not sure, read the appropriate sections before making your selection.

Life sciences study design

All studies must disclose on these points even when the disclosure is negative.

Sample size	Every experiments consists of at least 3 independent biological replicates to allow for statistical analysis
Data exclusions	No data were excluded from analyses
Replication	Each experiment was repeated a minimum of 2 times to determine reproducibility. Replication results were successful and the representative nature of data and number of independent repeats is mentioned in each figure legend.
Randomization	For in vivo experiments, treatments were randomised within and across cages of mice.
Blinding	For in vivo experiments, researchers were blinded to the treatment each animal received until after data were analyzed. Similarly, researchers were blinded to the genotype of animals in in vitro experiments comparing knockout and wildtype animals until after data were analyzed.

Reporting for specific materials, systems and methods

We require information from authors about some types of materials, experimental systems and methods used in many studies. Here, indicate whether each material, system or method listed is relevant to your study. If you are not sure if a list item applies to your research, read the appropriate section before selecting a response.

Materials & experimental systems

n/a	Involved in the study	
☐	☒	Antibodies
☒	☐	Eukaryotic cell lines
☒	☐	Palaeontology
☐	☒	Animals and other organisms
☐	☒	Human research participants
☒	☐	Clinical data

Methods

n/a	Involved in the study	
☒	☐	ChIP-seq
☐	☒	Flow cytometry
☒	☐	MRI-based neuroimaging

Antibodies

Antibodies used

Conjugated antibodies against the following antigens were used for murine samples: CD25 (Thermo Fisher Scientific, Waltham, MA, USA, Cat# 12-0251-82, RRID:AB_465607), CD3 (BioLegend Cat# 100335, RRID:AB_10898314), CD3 (BioLegend Cat# 100218, RRID:AB_1595492), CD4 (BD Biosciences Cat# 553051, RRID:AB_398528), CD4 (BioLegend Cat# 100414, RRID:AB_312699), CD44 (BioLegend Cat# 103020, RRID:AB_493683), CD44 (BioLegend Cat# 103047, RRID:AB_2562451), CD44 (BD Biosciences Cat# 553134, RRID:AB_394649), CD45.1 (BD Biosciences Cat# 612811, RRID:AB_2870136), CD45.2 (BD Biosciences Cat# 563051, RRID:AB_2737974), CD62L (BioLegend Cat# 104428, RRID:AB_830799), CD69 (BioLegend Cat# 104506, RRID:AB_313109), CD8 (BD Biosciences Cat# 565968, RRID:AB_2739421), FOXP3 (Thermo Fisher Scientific Cat# 12-5773-80, RRID:AB_465935), RORγt (BD Biosciences Cat# 562894, RRID:AB_2687545), GM-CSF (BioLegend Cat# 505406, RRID:AB_315382), IFNγ (Thermo Fisher Scientific Cat# 25-7311-82, RRID:AB_469680), IL-17a (BioLegend Cat# 506908, RRID:AB_536010), IL-17 (Thermo Fisher Scientific Cat# 17-7177-81, RRID:AB_763580), pSTAT3 (pY705) (BD Biosciences Cat# 612569, RRID:AB_399860), pSTAT6 (pY641) (Thermo Fisher Scientific Cat# 17-9013-42, RRID:AB_2573274), Conjugated antibodies against the following antigens were used for human samples: CD3 (BioLegend Cat# 317340, RRID:AB_2563408), CD4 (BioLegend Cat# 317432, RRID:AB_2028494), CD45RA (BioLegend Cat# 304128, RRID:AB_10708880), IFNγ (BioLegend Cat# 502515, RRID:AB_493029), IL-17a (BioLegend Cat# 512306, RRID:AB_961394)

For tissue culture the following antibodies were used:

Mouse: anti-CD3 (BioXcell Cat# BE0001-1), anti CD28 (BioXcell Cat# BE0015-1) anti-IFNγ and anti-IL-4 (BioXcell Cat # BE0055 and BE0045).

Human:anti-CD3 (BioLegend Cat# 317302, RRID:AB_571927) and 2 µg/mL anti-CD28 (BioLegend Cat# 302902, RRID:AB_314304) anti-IFNγ (BioLegend Cat# 502402, RRID:AB_315223)

Validation

Antibodies were validated by manufacturers as described on their websites

Animals and other organisms

Policy information about studies involving animals; ARRIVE guidelines recommended for reporting animal research

Laboratory animals

All mice used were between 8 and 14 weeks of age, with littermates used unless otherwise stated. Wild type CD57BL/6NCrl (MGI ID 2683688), MyD88-/-, TLR2-/- (Tlr2tm1Aki, MGI ID 2178675) and TLR4-/- mice (Tlr4tm1Aki, MGI ID 1860885) strains were used.

Wild animals	The study did not involve wild animals
Field-collected samples	The study did not involve samples collected in the field
Ethics oversight	Ethics approval for animal studies was granted by the Australian National University Animal Ethics and Experimentation Committee, in accordance with the National Health and Medical Research Council's Australian Code for the Care and Use of Animals for Scientific Purposes and the ACT Animal Welfare Act 1992.

Note that full information on the approval of the study protocol must also be provided in the manuscript.

Human research participants

Policy information about studies involving human research participants

Population characteristics	Samples were collected from both male and female healthy donors aged 20-35 years.
Recruitment	Donors were sourced from the staff members of the John Curtin School of Medical Research, ANU.
Ethics oversight	Experiments using human samples were approved by the Australian National University Human Research Ethics Committee.

Note that full information on the approval of the study protocol must also be provided in the manuscript.

Flow Cytometry

Plots

Confirm that:

- [x] The axis labels state the marker and fluorochrome used (e.g. CD4-FITC).
- [x] The axis scales are clearly visible. Include numbers along axes only for bottom left plot of group (a 'group' is an analysis of identical markers).
- [x] All plots are contour plots with outliers or pseudocolor plots.
- [x] A numerical value for number of cells or percentage (with statistics) is provided.

Methodology

Sample preparation	Murine cells were collected from spleens, lymph nodes, peyers patches and blood as stated in methods and figure legends. Organs were mashed through a 70uM strainer and cells isolated by MACS or further processed for ex-vivo analysis as specified in the methods.<br>Human blood was collected into either K2 EDTA or sodium heparin coated vacutainers (BD) and processed for NET-generation or cell sorting as stated in the methods section.
Instrument	Flow cytometry was performed using a LSRII machine (BD Biosciences). Imaging flow cytometry was performed using the Amnis Imagestream at 60X magnification.
Software	Flow cytometry data were analyzed using FlowJo 10.5.0 software (Tree Star). Imaging flow cytometry was analysed using IDEAS (version 6.2)
Cell population abundance	Purity of naïve CD4+ cells was verified to be above 99% in all cases. Purity was determined by analyzing the sorted sample by flow cytometry using the same settings as used for sorting the population.
Gating strategy	Specific gating strategies are described in the methods and in figure legends. All gating followed the basic pattern of lymphocytes (or debris exclusion for cultured cells)(FSC/SSC), doublet exclusion (FSC-A/FSC-H) followed by removal of dead cells (negativity for viability marker).

- [x] Tick this box to confirm that a figure exemplifying the gating strategy is provided in the Supplementary Information.
